# Ovarian ERβ cistrome and transcriptome reveal chromatin interaction with LRH-1

**DOI:** 10.1186/s12915-023-01773-1

**Published:** 2023-11-29

**Authors:** Madeleine Birgersson, Rajitha Indukuri, Linnéa Lindquist, Lina Stepanauskaite, Qing Luo, Qiaolin Deng, Amena Archer, Cecilia Williams

**Affiliations:** 1 Science for Life Laboratory (SciLifeLab), Department of Protein Science, KTH Royal Institute of Technology, 171 21 Solna, Sweden; 2 Department of Biosciences and Nutrition, Karolinska Institutet, 141 83 Huddinge, Sweden; 3 Department of Physiology and Pharmacology, Karolinska Institutet, 141 83 Huddinge, Sweden

**Keywords:** ChIP-seq, ERβ, Esr2, LRH-1, Nr5a2, Ovary, RNA-seq

## Abstract

**Background:**

Estrogen receptor beta (ERβ, Esr2) plays a pivotal role in folliculogenesis and ovulation, yet its exact mechanism of action is mainly uncharacterized.

**Results:**

We here performed ERβ ChIP-sequencing of mouse ovaries followed by complementary RNA-sequencing of wild-type and ERβ knockout ovaries. By integrating the ERβ cistrome and transcriptome, we identified its direct target genes and enriched biological functions in the ovary. This demonstrated its strong impact on genes regulating organism development, cell migration, lipid metabolism, response to hypoxia, and response to estrogen. Cell-type deconvolution analysis of the bulk RNA-seq data revealed a decrease in luteal cells and an increased proportion of theca cells and a specific type of cumulus cells upon ERβ loss. Moreover, we identified a significant overlap with the gene regulatory network of liver receptor homolog 1 (LRH-1, Nr5a2) and showed that ERβ and LRH-1 extensively bound to the same chromatin locations in granulosa cells. Using ChIP-reChIP, we corroborated simultaneous ERβ and LRH-1 co-binding at the ERβ-repressed gene *Greb1* but not at the ERβ-upregulated genes *Cyp11a1* and *Fkbp5*. Transactivation assay experimentation further showed that ERβ and LRH-1 can inhibit their respective transcriptional activity at classical response elements.

**Conclusions:**

By characterizing the genome-wide endogenous ERβ chromatin binding, gene regulations, and extensive crosstalk between ERβ and LRH-1, along with experimental corroborations, our data offer genome-wide mechanistic underpinnings of ovarian physiology and fertility.

**Supplementary Information:**

The online version contains supplementary material available at 10.1186/s12915-023-01773-1.

## Background

The highly regulated function of the ovary is essential for female fertility and endocrine homeostasis. The ovary is the source of oocytes and the major provider of the female steroid sex hormones estrogen and progesterone. Ovarian dysfunction is relatively common, with incidences of primary ovarian insufficiency and early menopause estimated at 3.7% and 12.2%, respectively [1]. Several factors are known to disturb ovarian functionality, including age [2, 3], diseases such as polycystic ovary syndrome [4], and lifestyle-related factors including obesity [5–9]. Yet, the exact mechanism behind ovarian dysfunction remains largely unknown.

Estrogen, along with progesterone, regulates female fertility. The production of estrogen is a cooperative interaction between theca and granulosa cells of the ovary. Theca cells produce androstenedione that diffuses to the neighboring granulosa cells where it is aromatized by aromatase (CYP19A1) to estrone (E1). E1 is then converted to 17β-estradiol (E2) by hydroxysteroid 17β dehydrogenase 1 (HSD17B1). Aromatase in the granulosa cells also converts the testosterone produced by the theca cells into E2. The effect of E1 and E2 is mediated through binding to the nuclear receptors estrogen receptor α (ERα, *ESR1*) and β (ERβ, *ESR2*), or the membrane G protein-coupled estrogen receptor 1 (GPER1). ERα is expressed in several female organs, including theca cells of the ovary, as well as in the hypothalamus, and the pituitary gland [10–12], and is essential for female reproduction. Women born with an inactivating mutation in this gene exhibit delayed puberty, primary amenorrhea, multiple ovarian cysts, and infertility [13, 14]. Mice lacking ERα are also infertile and develop cystic follicles [15, 16]. ERβ, which is a homolog of ERα, has a noticeably more restricted expression pattern and is predominantly expressed in the granulosa cells of the ovary [17–21]. Its function is also less understood. Only one woman has been described to be born with a dominant negative ERβ mutation. This woman was infertile with complete ovarian failure (undetectable ovaries) and reduced estrogen levels [22]. Female rats that lack ERβ develop ovaries but are infertile and lack an estrous cycle, have lower E2 levels in serum, and do not respond to gonadotropins [23]. A resulting activation of primordial follicles has been reported to lead to premature ovarian senescence in these females [24]. ERβ knockout (ERβKO or BERKO) female mice also develop ovaries, but lack follicular and oocyte maturation, and are subfertile [25]. They exhibit a reduced luteinizing hormone (LH) surge and reduced response to follicle-stimulating hormone (FSH) [25, 26], reduced estrogen surge at diestrus [27, 28], and they become infertile by 6 months of age [27]. This phenotype has been experimentally demonstrated to be dependent on ovarian ERβ, as it can be rescued by transplantation of a wild-type (WT) ovary into knockout mice [28]. Previous microarray and RNA-sequencing (RNA-seq) comparisons of granulosa cells from WT and ERβKO rodents, identified differences in FSH and LH target gene expression (e.g., *Lhcgr*, *Cyp11a1*, *Cyp19a1*, *Runx2*, and *Ptgs2*), confirming the significance of ERβ during folliculogenesis and ovulation [29, 30]. However, the exact mechanism of ERβ in the ovary, including its genome-wide endogenous chromatin binding, cross talk, and direct transcriptional impact has not been determined [31].

An obstacle for ERβ research has been a lack of specific antibodies [17]. However, in recent years, a validated, specific antibody has become available [17, 19, 21, 32–34]. In our study, this antibody was used to provide new insight into the role of ERβ in normal ovarian function and fertility. We performed chromatin immunoprecipitation sequencing (ChIP-seq) of ERβ in mouse ovaries to provide a map of endogenous ERβ chromatin-binding sites. We further compared its cistrome with corresponding RNA-seq data (WT versus ERβKO ovaries) and describe the direct role of ERβ in the regulation of specific ovarian functions. Finally, we demonstrate crosstalk between LRH-1, which is essential for ovulation, and ERβ, a finding that progresses our understanding of the molecular mechanism underlying female fertility.

## Results

### ERβ is expressed in granulosa cells throughout folliculogenesis

**Fig. 1:**
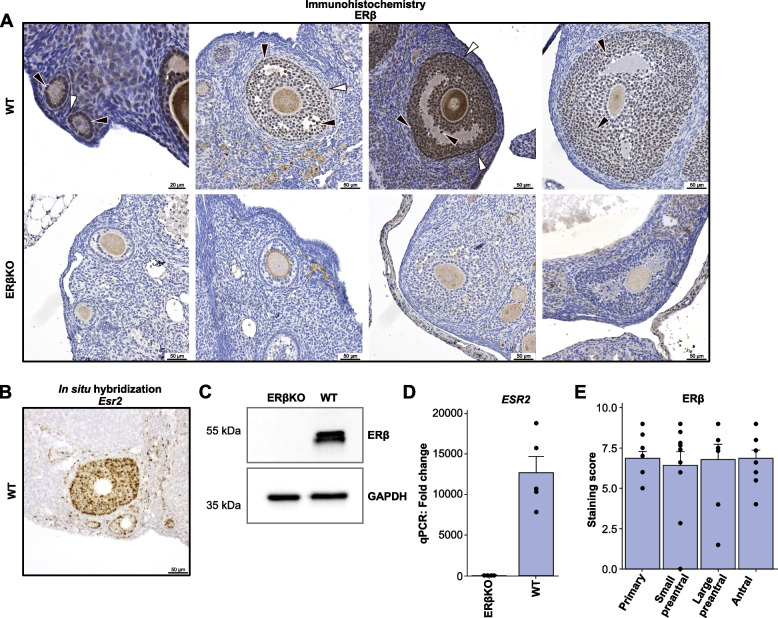
ERβ is expressed in granulosa cells and some stromal cells of the ovary. **A** IHC with the validated antibody PPZ0506 in WT (upper panel, bar indicates 20 μm in the left panel and 50 μm in all other, black arrows indicate granulosa cells and white theca cells, *n* = 4) and ERβKO (lower panel, *n* = 4) mouse ovary. **B** In situ hybridization with a probe against *Esr2* in WT female ovary (*n* = 2 mice, one representative ovary shown in image) confirms ERβ expression in granulosa cells, in cells surrounding the outer layer of theca cells, and in some stromal cells in mouse ovary. **C** Western blot (one of 2 replicates, each with 2 pooled ovaries), and **D** qPCR analysis (*n* = 5, paired t-test) corroborates the loss of ERβ expression in ERβKO ovaries. Both mouse ERβ isoforms (549 aa, calculated molecular weight 61.2 kDa and 567 aa ERβ_ins with 18 inserted aa,, calculated molecular weight 63.2 kDa) are visible near the expected sizes, with both bands being absent in the knockout ovary. **E** Assessment of ERβ protein expression by IHC in WT mice (*n* = 11), separated by follicular stage and each follicle scored according to staining intensity and area

ERβ is known to be expressed in granulosa cells, but its expression in other cells including theca cells has been debated [19]. We performed immunohistochemistry (IHC) with an ERβ antibody (PPZ0506) that has been thoroughly validated across different tissues in humans and rodents [17, 19–21]. We confirmed that ERβ was strongly expressed in the nucleus of granulosa cells. Some nuclear staining was further observed in cells surrounding the theca layer and in a few cells in the stroma, but not within the theca cell layer itself (Fig. 1A, upper panel). In ERβKO ovaries, ERβ expression (IHC) was absent from the granulosa cells (Fig. 1A, lower panel). However, some cytoplasmic staining, mostly of stroma cells, was still present and was deemed to be non-specific. We corroborated the ERβ expression using ERβ RNA in situ hybridization. This confirmed its high expression in granulosa cells and showed clear evidence of ERβ in the layer surrounding the theca cells of the follicle, which may correspond to the follicular microvasculature, and in a minority of other stromal cells, while the theca cells themself appeared blank (Fig. 1B). ERβ expression was further corroborated by western blot (WB) using the same antibody and by qPCR directed towards the exon deleted in the knockout (Fig. 1C-D). The WB of the WT ovary reveals two bands of similar intensity near the expected size. These are likely to correspond to the two murine splice variants of ERβ, the 567 amino acid (aa) ERβ_ins (isoform 1 / NM_207707.1) and the 549 aa ERβ (isoform 2 / NM_010157.3) which differ by an 18-aa long sequence (approx. 2 kDa) of the ligand binding domain. The epitope for the antibody is at the N-terminal part of the receptor (corresponding to the first coding exon), and the antibody thus recognizes both splice variants. The expression of the two splice variants was confirmed at the gene expression level (Additional file 1: Fig. S1). To be noted, the knockout mice have exon 3 deleted which creates a frameshift and premature termination during translation. Had any truncated proteins or peptides been expressed, these should have been recognized by the antibody. Since the granulosa cells of the knockout were primarily negative by IHC, we determined that ovarian ERβ is fully absent following knockout. Finally, we investigated if ERβ protein expression differed depending on the follicular stage (primary, small preantral, large preantral, antral), but did not find a significant change (Fig. 1E). We conclude that ERβ protein is highly and consistently expressed in granulosa cells during folliculogenesis and that ERβ is also expressed in cells surrounding the theca cells.

### The endogenous ovarian ERβ cistrome

**Fig. 2:**
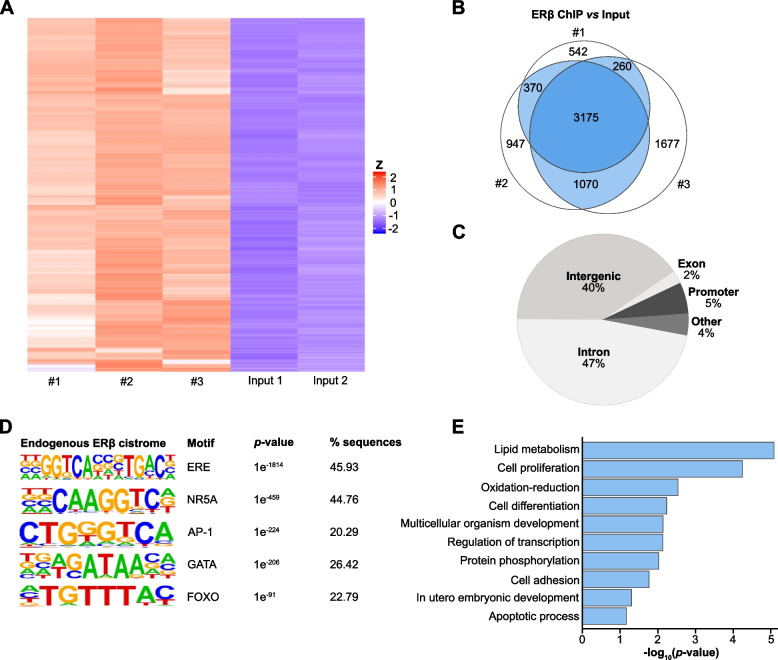
Genome-wide landscape of ERβ chromatin binding in mouse ovary. **A** ERβ chromatin-binding sites in mouse ovary per ChIP-seq replicate (*n* = 3 replicates, each with 14 pooled ovaries from 7 mice) in relation to corresponding input samples, visualized in a heatmap. **B** Venn diagram of detected ERβ-binding sites in ChIP-seq triplicates normalized against input. Sites present in at least two replicates, colored in blue, were used in further analysis. **C** The genomic distribution of ERβ-binding sites. All regions, except intergenic regions, were significantly enriched (*p* < 0.005). **D** Top-enriched DNA motifs among ERβ-bound genomic sequences, identified using HOMER de novo motif analysis (sorted by *p*-value). % sequences represent the percentage of ChIP:ed sequences (bound sites) that have a particular motif. The percentages will not add up to 100% as each sequence can have more than one motif. **E** Enriched biological functions among genes nearest ERβ chromatin-bound sites (within −50 kb to +2 kb)

ERβ is located within the nucleus (as can be noted in Fig. 1A) and functions as a transcription factor. To explore its endogenous genome-wide chromatin binding in the ovary, we performed ChIP-seq of WT mouse ovaries with the same ERβ antibody (PPZ0506) that was previously optimized for ChIP of human ERβ in cell lines [33, 34]. The experiment was performed in biological triplicates and compared to inputs. More than 80% of the produced sequencing reads were of high quality, and between 25 and 31 M reads per ChIP sample were aligned to the genome (GRCm38, Additional file 2: Table S1). A heatmap and Venn diagram illustrate that the majority of binding sites were detected in all three replicates (3175 sites, Fig. 2A-B). As many as 4875 ERβ-binding sites were detected in at least two ChIP replicates and were used for further analysis. The ERβ ChIP-seq was further validated by using ChIP-seq data from ERβKO ovaries instead of input (> 70% of sites confirmed, Additional file 1: Fig. S2A-B and Additional file 3: Table S2). In accordance with ERβ cistromes from other cell types [34–37], the majority of binding sites were located in introns and intergenic regions (87%), and a minority (5%) in promoter regions (-1 kilobase (kb) to +100 base pairs (bp) from the gene transcription start sites, TSS, Fig. 2C). Notably, the intronic binding sites were most frequently located in intron 1 (36%) or intron 2 (18%).

We next determined which transcription factor binding sequence motifs were significantly enriched in the ERβ-bound DNA. Using HOMER, we identified that the estrogen response element (ERE) was the most enriched (*p* = 1e^−1814^) and abundant motif (present in 45.9% of the bound sequences whereas the background frequency is 3.6%), as would be expected (Fig. 2D). Further, motifs of the well-known ER-tethering and pioneering factors AP-1, GATA, and FOXO were highly enriched (Fig. 2D). This confirmed the specificity of the antibody and the accuracy of the experiment. Interestingly, the NR5A motif was the second most enriched motif and as abundant (44.8% of bound sequences, significant enrichment over the 16.6% background frequency, *p* = 1e^−459^) as the ERE. The NR5A motif can be bound by two nuclear receptors: the steroidogenic factor 1 (SF-1/Nr5a1) and the liver homolog 1 (LRH-1/Nr5a2). Both are expressed in the mouse ovary and essential for ovulation [38, 39]. While SF-1 has been observed in relation to ERβ previously, an interaction with LRH-1 has not been reported. We found no difference in the types of motifs bound in promoter regions versus more distant (enhancer) regions (Additional file 1: Fig. S2C).

Finally, we mapped which genes’ TSS were located closest to each ERβ-binding site (Additional file 3: Table S2). Biological pathway analysis on those genes’ functions revealed enrichments related to lipid metabolism, cell proliferation, cell differentiation, multicellular organism development, cell adhesion, transcription regulation, and apoptosis (Fig. 2E). These are functions that ERβ is known (transcription regulation, cell proliferation, apoptosis, cell adhesion) or proposed (cell differentiation, multicellular organism development, lipid metabolism) to modulate in various cell types. This significant enrichment suggests that ovarian ERβ has the potential to impact these functions, although not all bound genes may be de facto regulated. Thus, we here describe the complete endogenous ovarian ERβ cistrome for the first time.

### ERβ influences the ovarian transcriptomic landscape

**Fig. 3:**
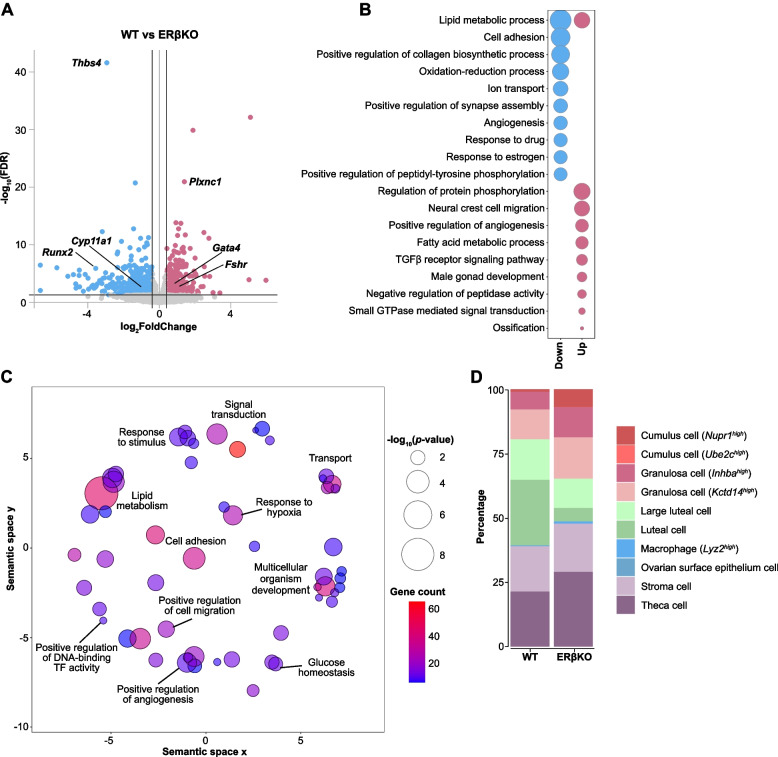
Impact of ERβ on the ovarian transcriptional landscape. **A** Volcano plot of ERβ-regulated genes. Genes were considered differentially expressed when FDR < 0.05 and log_2_FC >|0.4| (*n* = 5 WT, *n* = 4 ERβKO). **B** Top-10 enriched biological pathways of the down- (blue) and upregulated (red) DEGs. The size of the bubbles corresponds to the enrichment score (-log_10_(*p*-value)). **C** All significantly enriched biological pathways visualized in semantic space. The size and color of the bubbles correspond to the enrichment score (-log_10_(*p*-value)) and gene count, respectively. **D** Bar chart presenting the projected cell type abundance in the ovary of WT and ERβKO mice (Wilcoxon rank-sum exact test)

To map the consequences of ERβ deletion on the ovarian transcriptional landscape, we performed RNA-seq on ovaries from WT (*n* = 5) and ERβKO (*n* = 4) mice. We identified 803 differentially expressed genes (DEGs) following the loss of ERβ, with a relatively uniform distribution between up- and downregulated genes (375 upregulated and 427 downregulated, Fig. 3A, Additional file 4: Table S3). Thrombospondin 4 (*Thbs4*, adhesion) was the most significantly downregulated gene following loss of ERβ (Fig. 3A). *Thbs4* is abundant in normal ovary and related to the polycystic ovary syndrome-associated gene *Thbs1* [40]. Further, as would be expected, genes involved in response to estrogen were significantly enriched and specifically downregulated (incl., *Star*, *Igfbp2*, *Tgfbr1*, *Pdgfrb*) in the absence of ERβ (Fig. 3B, Additional file 4: Table S3). Other enriched functions among the downregulated genes included cell adhesion (e.g., *Thbs4*, *Itga1*, *Itga2*, *Itga4*, *Itga5*, *Cdh11*), oxidation-reduction (e.g., *Cyp11a1*, *Hsd17b7*), and ion transport (incl. potassium channels *Kcnab3*, *Kcnd2*, *Kcnj16*, *Kcnk3*, *Kcnma1*) (Fig. 3B). The ovarian expression of these genes is thus indicated to be upregulated as a consequence of ERβ expression. Among the genes upregulated in absence of ERβ (i.e., downregulated as a consequence of ERβ expression), we find FSH and LH targets (incl. *Fshr*, *Cyp11a1*, *Gata4*, *Runx2*) (Fig. 3A), along with genes related to neural crest cell migration (*Sema3b*, *Sema3c*, *Sema3e*, and *Sema3g*), fatty acid metabolism (e.g., *Fasn*), TGFβ receptor signaling (e.g., *Smad6*), and male gonad development (e.g., *Inha*, *Kitl*, *Gata1*, *Gata4*) (Fig. 3B). Moreover, the male sex determination gene desert hedgehog (*Dhh*) was upregulated. We further noted upregulation of plexin C1 (*Plxnc1*) in ERβKO ovaries (Fig. 3A). *Plxnc1* is related to *Plxnb1* which is involved in mouse follicular development [41]. Interestingly, *Greb1*, which is upregulated both by ERα [42] and ERβ (engineered expression, [43]) in human breast cancer cells, was found among these genes. These functions and genes are thus potentially repressed by ERβ in the ovary. Genes with functions in lipid metabolism (e.g., *Fads6* down, *Lep* up) and angiogenesis (e.g., *Angpt1* down, *Angpt2* and *Angpt4* up) were enriched among both up- and downregulated genes. When all DEGs (regardless of direction) were analyzed for biological process enrichment, lipid metabolism, glucose homeostasis, response to hypoxia, response to stimulus, and multicellular organism development were among the enriched functions (Fig. 3C, Additional file 4: Table S3). We conclude that deletion of ERβ impacts several pathways essential for normal ovarian function, including the repression of male gonad development.

### ERβ impacts the ovarian cell composition

Since the analysis above identified the gene expression of the complete ovary, we next used the RNA-seq data to investigate whether the loss of ERβ impacted the ovarian cell composition. By applying digital cytometry, using gene signatures from published mouse ovary single-cell RNA-seq data [44] along with CIBERSORTx [45], on our bulk RNA-seq data, we could estimate the abundance of different cell types. This identified in total 10 different cell types encompassing two types of granulosa cells (*Inhba*^high^ and *Kctd14*^high^), two types of luteal cells (regular and large), theca cells, two types of cumulus cells (*Nupr1*^high^ and *Ube2c*^high^), ovarian surface epithelial cells, macrophages (*Lyz2*^high^), and stromal cells. Following the loss of ERβ, a significant decrease in luteal cells, along with an increase of theca and *Nupr1*^high^ cumulus cell populations were indicated (Fig. 3D). The decrease in luteal cells confirms previous histological observations and can be directly linked to the impaired ovulatory phenotype that results in a reduced formation of corpus luteum in the ERβKO mice. Overall, our transcriptome analysis demonstrates a clear impact of ERβ on the cellular composition of the ovary.

### Characterizing ovarian ERβ target genes

**Fig. 4:**
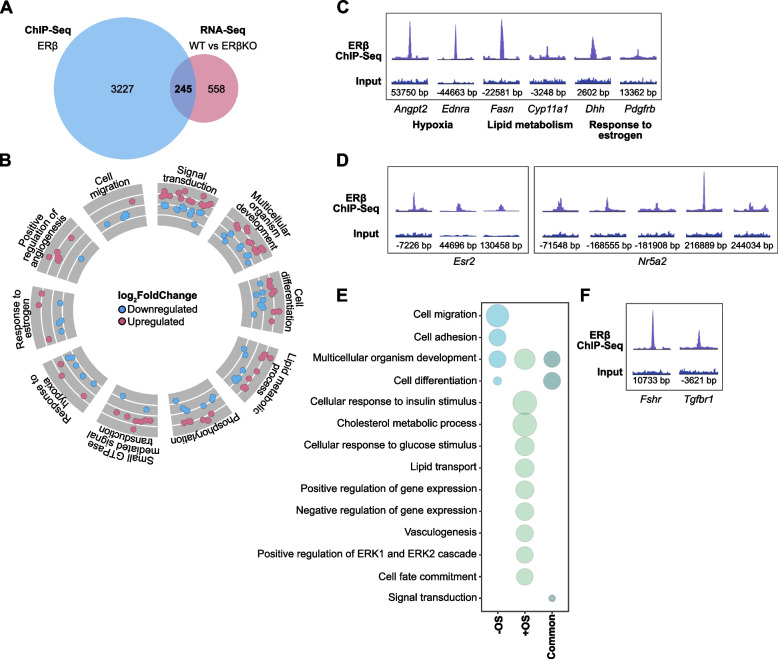
Identification of direct ERβ-regulated genes and biological pathways in mouse ovary. **A** Venn diagram representing the genes overlapping between ERβ ChIP-seq in WT ovaries (*n* = 3 replicates, each with 14 pooled ovaries from 7 mice) and RNA-seq performed in WT (*n* = 5) and ERβKO ovaries (*n* = 4). **B** Circle plot representing the top-10 enriched biological functions of the 245 overlapping genes between ChIP-seq and RNA-seq (from A). For each biological function, up- (red) and downregulated (blue) genes are represented. **C** ChIP peaks show enrichment of ERβ at chromatin by genes involved in specific functions. **D** ChIP enrichment at binding regions nearest to *Esr2* and *Nr5a2*. All tracks are set to the same Y-axis height for the ChIP-seq and input. **E** The top-10 enriched biological functions of the regulated genes identified by both ChIP-seq and microarray (before and after ovulatory signal, from [29]). The size of the bubbles corresponds to the enrichment score (-log_10_(*p*-value)). **F** ERβ ChIP signal at *Fshr* and *Tgfbr1*

In an effort to identify the direct ERβ transcriptional target genes in the ovary, we integrated our RNA-seq and ChIP-seq data. This revealed that as much as a third (30%, or 245 out of 803 genes) of the ERβKO ovarian DEGs were located nearest (their TSS) to one or multiple ERβ-binding chromatin sites (Fig. 4A). Assuming a connection between the ERβ chromatin binding and the subsequent transcript regulation upon its loss, we here denote these 245 genes as direct transcriptional targets of endogenous ERβ in the ovary. A slightly larger proportion of these binding sites (52%) contained an ERE motif compared to all ERβ-bound sites (46%). The direct targets were regulated in both directions following the deletion of ERβ (49% up and 51% down). Again, the direct target genes were enriched for functions related to response to estrogen (e.g., *Dhh*, *Pdgfrb*, *Gata4*), lipid metabolism (e.g., *Fasn*, *Cyp11a1*), positive regulation of angiogenesis (e.g., *Angpt2*, *Angpt4*), cell differentiation (e.g., *Etv6*), and multicellular organism development (e.g., *Cebpa*, *Pak3*, *Fzd1*, *Greb1*), (Fig. 4B). Also, response to hypoxia (e.g., *Angpt2*, *Endra*) was among the most enriched functions of the target genes (Fig. 4B). Related ERβ chromatin binding is exemplified in Fig. 4C. It can be noted that overall, few of the directly regulated genes had a binding site in the promoter region (13 out of the 245 genes, or 5%), encompassing *Inha*, *Hsd17b1*, *Gata4*, *Neat1* (long non-coding RNA), *Cpm*, *Epb41l2*, *Mylk3*, *Mdfic*, *Zfp219*, *Epb41l1*, *Pik3cd*, *Skil*, and *Fosl2*. Notably, the majority of the direct targets (158 out of 245 or 64%) had an ERβ binding site in an intron, most often (86 genes, or 35%) in intron 1. This included *Greb1* (4 binding sites in intron 1), *Angpt4*, *Pak3* and *Bcl2* (each 2 binding sites in intron 1), and *Dhh* and *Pdgfrb* (each 1 binding site in intron 1). Thus, a primary mechanism whereby ERβ regulates genes appears to involve its binding to intron 1.

Since nuclear receptors may crosstalk at several levels (regulation, interaction, shared transcriptional targets), we next explored our datasets to identify potential nuclear receptor regulation by ERβ. We found that the TSS of multiple nuclear receptor genes were located closest to several ovarian ERβ-binding sites and could thus be potential direct targets (Additional file 5: Table S4). This included ERβ itself (*Esr2*, three binding sites visualized in Fig. 4D) and LRH-1 (*Nr5a2*, five binding sites, Fig. 4D), indicating a potential regulatory loop. Similarly, SF-1 (*Nr5a1*) had two intronic ERβ-binding sites. However, neither LRH-1 nor SF-1 transcripts were significantly regulated in the ERβKO ovary. The remaining nuclear receptor genes that were located by ERβ-binding sites (ERα, PR/*Pgr*, SHP/*Nr0b2*, PXR/*Nr1i2*, GR/*Nr3c1*, NUR77/*Nr4a1*, COUP-TF-I/*Nr2f1*, and COUP-TF-II/*Nr2f2*), as well as several nuclear receptor coregulators (incl. *Nrip1*, SRC1/*Ncoa1*, and *Ncor*), were also not detected as significantly regulated in the knockout ovaries. This lack of transcriptional regulation is in line with the result generated by Binder and colleagues using isolated granulosa cells from WT and ERβKO ovaries, where only *Pgr* was differentially expressed following ERβ knockout after ovulatory stimuli [29, 46]. To conclude, although ERβ can bind to chromatin regions close to several nuclear receptors and potentially regulate them, we did not find evidence that it does so in the ovary.

Finally, to explore which direct targets may be most consequential for ovarian function, we searched for genes where ERβ would be essential for their expression in the ovary. That is, genes whose transcripts were near absent (< 2 Fragments Per Kilobase of transcript per Million mapped reads, FPKM) in the ERβKO ovary but at least 2-fold upregulated in the WT ovary, and with high confidence (FDR < 0.0001) across the individual samples. The expression of 14 genes fulfilled these strict requirements (Table 1). Eight of these had an ERβ chromatin-binding site, nearly all (7/8) located in an intron. Interestingly, a majority (9 out of 14, or 64%) encoded for proteins located in membranes such as cell surface receptors for low density lipoprotein (Lrp8, Lrp11) and catecholamine (Adrb2). Thus, the proteins that appear to be most dependent on ERβ for their ovarian expression are located in cellular membranes with roles in cell signaling, and they are regulated by ERβ through intronic chromatin binding. Taken together our study identifies direct targets of ERβ which furthers our understanding of its role in the ovary.

**Table 1:** Ovarian genes that appear dependent on ERβ for their in vivo expression

**Gene**	**Gene name**	**log**_**2**_**FC**	**ERβKO (FPKM)**	**WT (FPKM)**	**ERβ-binding site**
*Bhmt*	betaine-homocysteine methyltransferase	-6,7	0,3	34,0	Intergenic
*Mmel1*	membrane metallo-endopeptidase-like 1	-5,1	0,3	9,1	-
*Ssu2*	ssu-2 homolog (C. elegans)	-4,2	0,3	6,2	-
*Slc38a3*	solute carrier family 38, member 3	-3,2	1,0	10,1	Intron 1
*Lrp8*	low density lipoprotein receptor-related protein 8, apolipoprotein e receptor	-3,1	1,5	13,4	Intron 1
*Them5*	thioesterase superfamily member 5	-2,3	0,4	2,2	-
*Cabp1*	calcium binding protein 1	-1,9	0,9	4,2	Intron 1
*Tspan11*	tetraspanin 11	-1,9	0,9	3,1	Intron 2
*Lrp11*	low density lipoprotein receptor-related protein 11	-1,6	1,9	5,8	Intron 1
*Abca7*	ATP-binding cassette, sub-family A (ABC1), member 7	-1,6	1,6	4,9	Exon
*Stx11*	syntaxin 11	-1,4	1,3	3,4	-
*Adrb2*	adrenergic receptor, beta 2	-1,3	1,3	3,0	-
*Ctnna2*	catenin (cadherin associated protein), alpha 2	-1,2	1,0	2,2	Intron 1
*Nipal1*	NIPA-like domain containing 1	-1,1	1,2	2,6	-

Genes that are not expressed (< 2 FPKM per RNA-seq) in ERβKO ovary but expressed (> 2 FPKM and at least 2-fold more) in WT ovary, with high confidence of regulation across the individual samples (FDR < 0.0001). FPKM values represents average ovarian expressions (WT: *n* = 5 mice; ERβKO: *n* = 4 mice)

### ERβ gene regulation in granulosa cells during the ovulatory process

Previously, microarray comparisons of granulosa cells collected from large antral follicles of WT and ERβKO mice, before and after ovulatory signal, have identified 1361 genes related to ERβ expression [29, 46]. Only 5% (70 genes) of those were detected both before and after ovulatory signaling (Additional file 1: Fig. S2D). Since ERβ is primarily expressed in granulosa cells, we compared this data with our ERβ results from the whole ovary. While only 12% (164 of 1361) of the genes detected as regulated in the microarray were differentially expressed also in the WT and ERβKO ovarian RNA-seq (Additional file 1: Fig. S2D), the exact same proportion of genes (30%, 413 of 1361 vs 245 of 803 genes detected by RNA-seq) had an ERβ chromatin-binding site per our cistrome (Fig. 4A and Additional file 1: Fig. S2D). Exploring the pathway enrichment of all ERβ-bound (per our ChIP-seq) microarray-detected genes (413) showed that, similarly to our ERβ-bound genes in RNA-seq, developmental and differentiation genes were regulated both before and after ovulatory signal whereas other pathways were restricted to either condition (Fig. 4E). For example, genes involved in cell migration and cell adhesion were primarily regulated before the induction of ovulation, while target genes with functions in response to insulin, and cholesterol or lipoprotein metabolic processes were regulated after ovulatory signal. Genes with an ERβ chromatin-binding site, whose expression were detected as regulated in both studies (77 genes), included the key ovarian genes *Dhh* (Fig. 4C), *Fshr*, and *Tgfbr1* (Fig. 4F). Thus, several of the same genes and functions were detected in both studies, but some (including response to estrogen and angiogenesis) were only detected in our in vivo study.

### Conserved ERβ regulation between species

To explore a potential relation to human health, we compared the genes located by ERβ-bound sites in the mouse ovary (endogenous ERβ) with those previously assessed in human cell lines (only ERβ ChIP-seq data from exogenous ERβ in other cell types than ovary were available), and performed with the same validated antibody [34, 47]. This analysis showed that over one-third (1332 of 3472, or 38%) of the genes bound by ovarian ERβ (i.e., whose TSS was the closest located to the bound sites) in the mouse genome, were also bound by ERβ in human cells (Additional file 6: Table S5). Moreover, most of these genes were bound in their intronic regions in both species. In contrast, the genes bound in their promoter, transcription termination site (TTS), exon, 3’ or 5’ UTR regions, rarely had such conserved location. We also note that most of the binding sites in genes bound by ERβ in both species (1332 genes, corresponding to 2038 linked binding sites in mice) had an ERE motif (1138 of 2038, or 56%), and a nearly as large proportion (923 of 2038, or 45%) contained the NR5A motif, most commonly in combination with an ERE. Thus, also in the conserved genes, both ERE and NR5A were more frequent than the occurrence of the pioneering GATA motif (561 sites). When we specifically looked at the ovarian direct target genes (the 245 genes that were also regulated at the transcript level in the mouse), more than half of them (54% or 132 genes) also exhibited an ERβ binding site in the human cells, again most commonly in the introns (72 out of 132 genes). Here, however, the conservation was strong for the few promoter-bound target genes (mouse ovary) where nearly all (11/13 genes or 85%) were bound by ERβ in humans too, albeit only three (*INHA*, *EPB41L2*, *SKIL*) were bound at the promoter also in humans. These three direct targets with conserved promoter binding were either upregulated (*Skil* and *Epb41l2*) or repressed (*Inha*) by ERβ in the mouse ovary (Additional file 6: Table S5). Finally, we compared our cistrome and transcriptome to human granulosa-enriched genes from the Human Protein Atlas [48]. Out of the genes defined as granulosa-enriched in humans, over a quarter (27%, or 133 of 496 genes) were either bound by ERβ (mouse or human) and/or regulated following ERβ knockout (mouse ovary) in our data sets (Additional file 6: Table S5). Twenty-four of these genes were bound and regulated (here denoted direct ERβ targets) in the mouse ovary (incl. *DHH*, *FSHR*, *GATA4*, *GREB1*, *HSD17B1*, *INHA*, *INHBB*, *LRP5*, and *LRP8*). Thus, although there are major species differences between human and murine fertility, the chromatin binding of ERβ on key targets is relatively conserved. This indicates that key ovarian genes (e.g., *INHA*, *EPB41L2*, and *SKIL*) and transcription factors (e.g., *GATA6*, *LRH-1*, and *PPARG*) are of particular importance for ERβ-mediated female fertility also in humans.

### ERβ may co-regulate targets with LRH-1 in granulosa cells

**Fig. 5:**
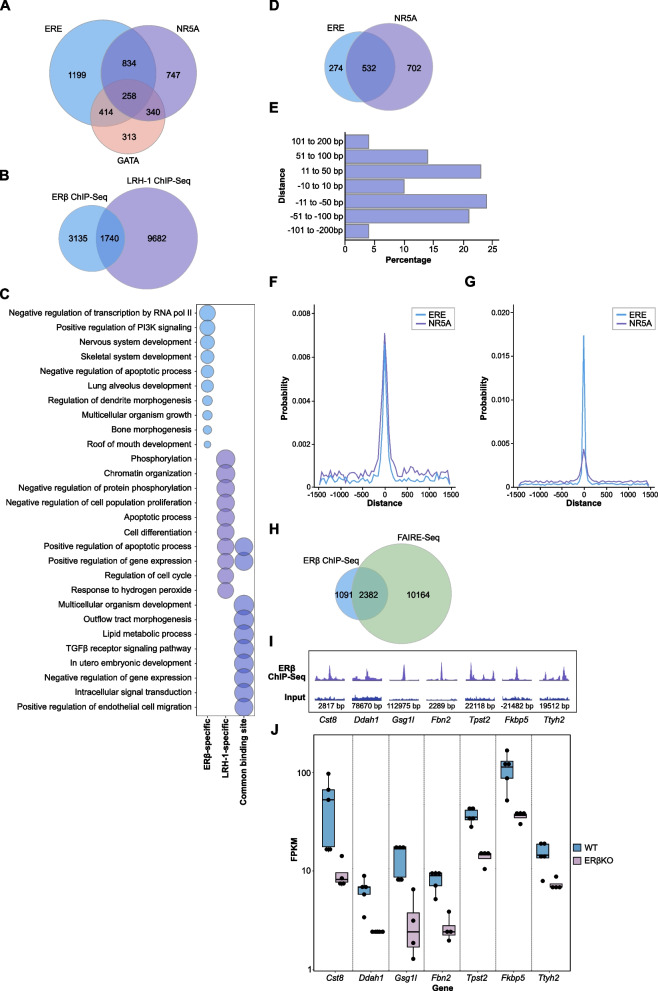
ERβ shares ovarian cistrome with LRH-1. **A** Venn diagram comparing distribution and co-occurrence of ERE, GATA, and NR5A motifs in all identified ERβ-bound sequences. **B** Venn diagram representing the overlapping chromatin-binding sites between ERβ and LRH-1. **C** Enriched biological pathways, summarized by REVIGO, comparing genes located nearest to chromatin bound by both ERβ and LRH-1 or specifically bound by either ERβ or LRH-1. The size of the bubbles corresponds to the enrichment score (-log_10_(*p*-value)). **D** Venn diagram comparing distribution and co-occurrence of ERE and NR5A motifs in the the sequences bound by both ERβ and LRH-1. **E** The distance between ERE and NR5A motifs in the sequences containing dual motifs and bound by both ERβ and LRH-1 (from D) plotted within +/- 200 bp. **F**, **G** Density plots representing motif occurrence within +/- 1500 bp distance of **F** all common ERβ and LRH-1 binding sites, and **G** all ERβ-binding sites. **H** Venn diagram comparing ERβ ChIP-seq with accessible chromatin (genes) using publicly available FAIRE-seq data [51] from granulosa cells during ovulatory stimulation. **I**, **J** ChIP enrichment and corresponding gene regulation (RNA-seq) of critical genes that require ERβ. All tracks are set to the same Y-axis height for the ChIP-seq and input

Two species-conserved ERβ-bound genes of particular importance, LRH-1 and SF-1, are nuclear receptors that bind the DNA motif (NR5A) that we found enriched at a considerable fraction (44%, *p* = 1e^−459^, Fig. 2D). This suggests that ERβ may regulate these nuclear receptors and also function in the same chromatin-bound complex as either of them. In other tissues, these transcription factors are known to recruit, and thereby enable the function of, other nuclear receptors. For example, LRH-1 enables the function of FXR in the liver [49], and SF-1 can recruit DAX-1 (Nr0b1) to promoters in the adrenal gland [50]. Exploring all ovarian ERβ-bound sequences for presence of ERE, NR5A, or GATA (pioneering factor) motifs, we found that most (4105 out of 4875, 84%) harbored at least one of these motifs (Fig. 5A). Among the sequences harboring only one of these three motifs, ERE was predominant (1199), followed by NR5A (747) which was about twice as common as GATA (313). The combination ERE and NR5A at one single binding site (1092) was more prevalent than ERE and GATA (672). That the NR5A motif was more common than the pioneering factor GATA motif could indicate that an NR5A nuclear receptor may co-regulate ovarian genes with ERβ and/or bind first and bring ERβ in. To investigate if the identified NR5A motifs were de facto bound by an NR5A nuclear receptor (LRH-1 or SF-1) in the ovary, we used a publicly available LRH-1 ChIP-seq data set from isolated mouse granulosa cells (GSE119508, [51, 52]). We investigated whether LRH-1 chromatin-binding sites overlapped with ERβ-binding sites within the ChIP-seq peak sizes (200 nucleotides, nt). We identified that over a third of all ERβ-bound sequences (1740 out of 4875, 36%) were indeed de facto also bound by LRH-1 in granulosa cells (Fig. 5B, Additional file 7: Table S6). This demonstrates that LRH-1 and ERβ, to a large extent, bind the same (200 nt) regulatory chromatin sites in the ovarian genome. This supports the interpretation that LRH-1 is important for ERβ function in the ovary, or vice versa.

Next, we explored potential differences in function (enriched biological pathways) between genes co-bound by ERβ and LRH-1 and those bound by only one receptor. To condense the list, all biological pathways were run through REVIGO (top-ten functions illustrated in Fig. 5C). We determined that genes bound by either nuclear receptor (whether alone or together) were strongly enriched for genes involved in apoptosis. The ERβ-only bound genes were specifically enriched for PI3K signaling, extracellular matrix organization, fertilization, ERK1/ERK2 cascade, male gonad development, and positive regulation of NFκB transcription factor activity. The LRH-1-only pathways, on the other hand, were strongly enriched for chromatin organization and cell differentiation. Finally, the co-bound genes had multicellular organism development as the most enriched function (ERβ-only and LRH-1-only were also enriched for various developmental genes) and were uniquely enriched for lipid metabolic process and the TGFβ receptor signaling pathway. We thus note clear differences in functions of the genes that ERβ and LRH1 both bind (at close distance), compared to those bound only by one of the receptors.

### ERβ-LRH-1 chromatin interactions

The co-binding of two ovulation regulators, ERβ and LRH-1, to the same ovarian DNA locations, points in the direction that this is a key ovarian molecular mechanism. To better understand this mechanism, we questioned whether LRH-1 might recruit ERβ (bound DNA harbors only NR5A motif), if ERβ might recruit LRH-1 (only ERE motifs), if both may bind simultaneously, or if they compete. In an effort to explore this, we first investigated to which extent the de facto dual-bound sequences (approx. 200 nt) harbored both NR5A and ERE motifs. We found that the largest group (702 out of 1740 de facto dual bound sites, or 40%) harbored an NR5A but not an ERE motif. The second largest group (532 sites, 31%) held both an ERE and an NR5A motif (Fig. 5D), whereas the smallest group (274 sites, 16%) had an ERE without an NR5A motif (the remaining 13% lacked both two motifs). Altogether, this may indicate that LRH-1 first binds to the NR5A motif and brings ERβ into the complex.

However, a second mechanism is possible for a sizeable fraction (31%) of chromatin events where both motifs are present: LRH-1 and ERβ either bind adjacent to each other or compete for binding. To explore the latter question, we first investigated the distance between the dual ERE and NR5A motifs (532 sites de facto bound by both receptors, although not necessarily at the same time). We found that in nearly all cases (92%), the two motifs were within 100 bp of each other (Fig. 5E). Further, the density plot clearly shows that both motifs had an equally high probability occurrence within a 500 bp distance of the binding site for these dual-bound sequences (Fig. 5F). This is to be compared with the density plot for all ERβ-binding sites where the ERE has the highest probability occurrence, and NR5A comes a distant second (Fig. 5G). A technical caveat is that the NR5A motif sequence overlaps with the complementary sequence of the ERE half-site motif with all but 3 additional nucleotides, making this analysis sensitive to artifacts. For this reason, we separated the sites where the NR5A motif directly or within 10 bases overlapped with an ERE motif and found that this only occurred for 88 out of the 532 sequences and did thus not impact the overall results. Evidently, the NR5A and ERE binding sites were commonly clearly separated, supporting that binding of the two nuclear receptors to their respective motifs near each other may occur.

**Fig. 6:**
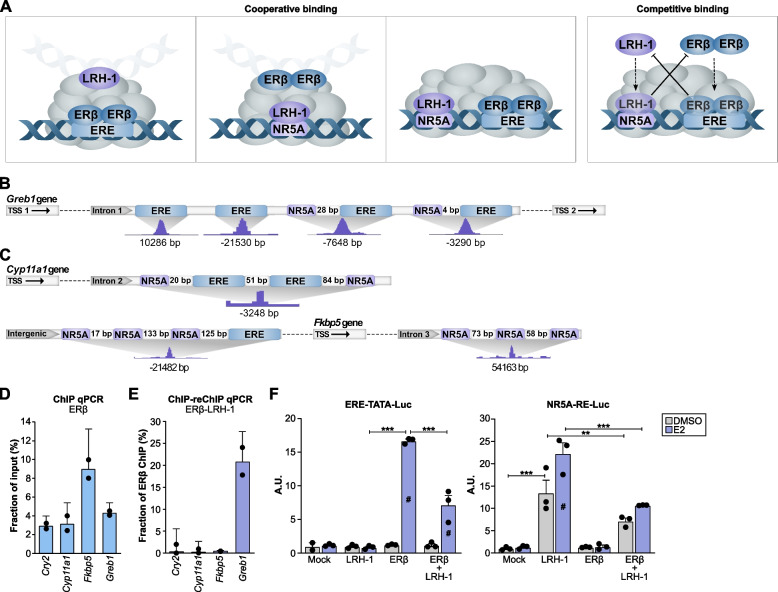
ERβ and LRH-1 are part of the same transcriptional complex. **A** Proposed binding mechanisms on chromatin bound by both ERβ and LRH-1. **B**, **C** Illustration of intergenic and intronic binding sites of ERβ, with corresponding ChIP-seq peak (below), for sites also bound by LRH-1, by genes **B ***Greb1*, with distant to *nearest* TSS (isoform 1, NM_015764, or isoform 2, NM_001252071) indicated, along with the location of ERE and NR5A motifs, and **C ***Cyp11a1*, and *Fkbp5*. **D** ERβ ChIP-qPCR (*n* = 1, with 8 pooled ovaries from 4 mice) confirms binding of ERβ to respective site, normalized to input. **E** ERβ-LRH-1 ChIP-reChIP followed by qPCR (one out of two experiments illustrated here, with 14 pooled ovaries from 8 mice, in technical duplicates) supports dual binding of ERβ and LRH1-1 to *Greb1* (intron 1), but not *Cyp11a1* (intron 2) or *Fkbp5* (intron 3) sites. Normalized to ERβ ChIP levels. **F**, **G** SW480 cells transfected to express ERβ and/or LRH-1, along with **F** ERE-TATA or **G** NR5A-RE luciferase reporter construct, show that ERβ and LRH-1 can repress each other’s transactivation activity (one out of three independent experiments per reporter construct illustrated here (*n* = 3), each with 3 technical replicates per condition, two-way ANOVA). # indicates significant difference by treatment (*p* < 0.05), ** *p* > 0.01, *** *p* < 0.001

Finally, we investigated to what extent the identified ERβ-binding sites were present in active chromatin regions. We compared our ERβ ChIP-seq data with Formaldehyde-Assisted Isolation of Regulatory Elements (FAIRE)-seq data performed in the granulosa cells under the same settings as the LRH-1 ChIP-seq [51]. We identified that 69% of the ERβ-bound genes were located nearby active chromatin regulatory regions (Fig. 5H). According to the FAIRE-seq data, the top motifs in the active chromatin regions were LRH-1 and CTCF, while ERE motifs represented 10% of all binding sites. Thus, as this shows that ERβ primarily binds within the active chromatin regions in granulosa cells, and a large proportion also harbors a NR5A motif and de facto LRH-1 binding sites, this further supports an active ERβ-LRH-1 co-regulatory mechanism in granulosa cell gene regulation. Potentially, ERβ and LRH-1 are part of the same transcriptional complex with a dual mode of interaction. LRH-1 may bring in ERβ to the chromatin complex, and they may co-regulate target genes. Alternatively, they may compete for binding at nearby motifs. Corresponding interpretations of potential molecular mechanisms are illustrated in Fig. 6A.

### Key ERβ-LRH-1 target genes

To explore which gene regulations this dual binding may be critical for, we scrutinized the genes with dual de facto binding of ERβ and LRH-1 (ChIP-seq), that were most strongly upregulated by ovarian ERβ (i.e., significantly higher expression in WT ovaries compared to ERβKO, using a more stringent cutoff of log_2_FC < -0.9, FDR < 0.05). This generated a list of 28 genes (Table 2), and corresponding ERβ binding and regulation are exemplified in Fig. 5I-J. To be noted, ERβ binds to the intron of half of these genes [14] and at multiple sites in nearly half [12] of the genes (*Ddah1* for example, has four ERβ binding sites, all located in intron 1). These genes are also, according to public databases, all expressed in granulosa cells in the mouse. Out of these 28 genes, 9 genes are bound by both receptors near each other at sites that harbor both an ERE and NR5A motifs (*Cst8*, *Gsg1l*, *Dpysl4*, *Fkbp5*, *Dsc2*, *Crybb1*, *Fbn2*, *Tpst2*, and *Ctnna2*) in at least one location. Among these genes, we find those encoding key developmental proteins such as germ cell-specific gene 1-like (*Gsg1l*, related to gonad development) and Fibrillin 2 (*Fbn2*, that sequester TGFβ in the extracellular matrix and impact osteoblast differentiation). Further, *Fkbp5*, known to be upregulated by several hormone receptors, has multiple ERβ and LRH-1 binding sites (Fig. 6C). *Fkbp5* (also known as FKBP54) is expressed both in mouse and human granulosa cells and encodes a co-chaperone that interacts with several hormone receptors (including AR, GR, and PR) and determines their responses [53]. Other significantly regulated genes with dual bindings (e.g., upregulated *Cyp11a1*, and downregulated *Greb1*) are also of interest (Fig. 6B-C). Both are highly expressed in mouse and human ovary. *Cyp11a1* acts upstream of the hormone biosynthetic process, and *Greb1* has been reported to be induced by E2 in granulosa cell tumors through an ERα-dependent mechanism [54]. We have previously demonstrated that *Greb1* is upregulated by both ERα and ERβ (exogenous) in breast cancer MCF-7 cells [43]. Taken together, our data reveal proposed ovarian target genes of a novel network interaction between ERβ and LRH-1 with potential implications for fertility.

**Table 2:** Top downregulated ERβKO ovary genes with de facto ERβ and LRH-1 chromatin binding

**Gene**	**Gene Name**	**log**_**2**_**FC**	**ERβ location(s)**	**TSS (kb)**	**ERE**	**NR5A**
*Slc38a3*	solute carrier family 38, member 3	-3.2	intron 4	9.1	-	-
*Lrp8*	low density lipoprotein receptor-related protein 8, apolipoprotein e receptor	-3.1	intron 2	9.0	-	+
*Cst8*	cystatin 8 (cystatin-related epididymal spermatogenic)	-2.3	intron 2	2.8	+	+
*Nrn1*	neuritin 1	-2.3	downstream gene^a^	224	-	+
*Sgk1*	serum/glucocorticoid regulated kinase 1	-2.1	intergenic	-38	-	+
*Gsg1l*	GSG1-like	-2.1	intron 2	113	+	×2
*Dpysl4*	dihydropyrimidinase-like 4	-2.1	upstream gene^b^	-62	+	+
*Cabp1*	calcium binding protein 1	-1.9	intron 1	2.7	-	-
*Tspan11*	tetraspanin 11	-1.9	downstream gene^c^	94	-	-
*Enpp6*	ectonucleotide pyrophosphatase/phosphodiesterase 6	-1.8	intergenic	-3.5	-	+
*Fkbp5*	FK506 binding protein 5	-1.7	1: intergenic	-22	+	×3
			2: intron 3	54	-	×3
*Dsc2*	desmocollin 2	-1.7	intron 1	0.7	+	+
*Crybb1*	crystallin, beta B1	-1.7	intergenic	-1.4	+	+
*Txndc2*	thioredoxin domain containing 2 (spermatozoa)	-1.6	1: upstream gene^d^	-62	-	×3
			2: intergenic	-5.3	-	+
*Fbn2*	fibrillin 2	-1.6	intron 2	2.3	+	+
*Lockd*	lncRNA downstream of Cdkn1b	-1.6	intergenic	25	-	×2
*Aif1l*	allograft inflammatory factor 1-like	-1.5	intron 2	4.5	+	+
*Parvb*	parvin, beta	-1.5	intron 1	36	-	+
*Tpst2*	protein-tyrosine sulfotransferase 2	-1.4	intron 1	22	+	×2
*Ddah1*	dimethylarginine dimethylaminohydrolase 1	-1.3	intron 1	47	+	-
*Mrap*	melanocortin 2 receptor accessory protein	-1.3	intergenic	-2.4	-	+
*Ctnna2*	catenin (cadherin associated protein), alpha 2	-1.2	1: intergenic	-192	×2	-
			2: intron 1	71	+	+
*Hao2*	hydroxyacid oxidase 2	-1.1	1: intergenic	-39	-	+
			2: intron 1	2.6	-	-
*Gpr37*	G protein-coupled receptor 37	-1.1	intergenic	239	-	×3
*Ttyh2*	tweety family member 2	-1.0	intron 3	16	-	×2
*Fzd1*	frizzled class receptor 1	-1.0	intergenic	262	-	+
*Nek6*	NIMA (never in mitosis gene a)-related expressed kinase 6	-1.0	intergenic	-13	-	-
*Slc6a6*	solute carrier family 6 (neurotransmitter transporter, taurine), member 6	-1.0	intergenic	-37	+	-

The list includes genes that, following loss of ERβ, are strongly downregulated (average log_2_FC < -0.9; FDR < 0.05) and are also nearest to both ovarian ERβ-bound and granulosa LRH-1-bound chromatin, co-bound within 200 bp. The presence of ERE and/or NR5A motif within the bound sequence is indicated by + or -, or if multiple, with the number of motifs present (×2 or ×3)

^a^Intron 5 of *Fars2* (non-significant trend of downregulation: log_2_FC: -0.26, FDR: 0.06)

^b^Intron 9 of *Jakmip3* (downregulated)

^c^Intron 2 of *Tspan9* (not significantly regulated)

^d^Intron 3 of *Rab31* (not regulated)

### ChIP-reChIP demonstrates interaction at select genomic fragments

To experimentally test whether ERβ and LRH-1 bind to the same regulatory DNA fragments simultaneously, we performed a ChIP-reChIP experiment. We explored DNA regions of target genes indicated to be bound by both receptors (*Greb1*, dual motifs in intron 1, -3290 bp from alternative TSS 2, see Fig. 6B; *Cyp11a1* dual motifs intron 2 and *Fkbp5* NR5A-only motif intron 3, Fig. 6C). Further, an ERβ-bound region not indicated to be bound by LRH-1 (the promoter-TSS of *Cry2*, where ERβ but not LRH-1 had a detected ChIP site) was included for comparison, as well as a gene desert where neither is expected to bind (negative control). Primers were designed to cover the selected areas with amplicons of approximately 100 bp, and qPCR on a 4^th^ ERβ mouse ovary ChIP experiment (average DNA size of 100–200 bp after sonication) confirmed ERβ binding at the identified sites and not at the gene desert (Fig. 6D). Next, two independent ERβ ChIPs were followed by LRH-1 reChIPs. Several controls were included. One control omitted the LRH-1 antibody and was used to ensure that ERβ-bound sequences were not re-IP:ed in the second step and also to confirm that unspecific enrichment to the affinity beads did not occur. qPCR of the eluted DNA of this control did not generate any amplification product which validated that there is no trace of unspecific binding of our target sequences. Secondly, we included the gene desert in the qPCR of the ERβ-LRH1 reChIP DNA to control for unspecific enrichment within our samples. This sequence did not amplify in the ChIP:ed samples, in line with our other controls. However, qPCR of the same samples revealed a substantial enrichment at the *Greb1* site but not at the *Cry2* site. This was true both when the results were normalized to input (fraction of input, Additional file 1: Fig. S3B) and when compared to corresponding ERβ ChIP levels (Fig. 6E and Additional file 1: Fig. S3A). This demonstrates that ERβ and LRH-1 simultaneously bind this intron 1 region of the *Greb1* gene (with ERE and NRA5 motifs). Further, while not enriched compared to *Cry2*, amplification products were detected for the *Fkbp5* (and *Cry2*) sites, but not at the *Cyp11a1* site. This may indicate that only one of the receptors, ERβ or LRH-1, binds at one time and that they may compete for binding at these sites. However, we cannot exclude that co-binding may occur to a lesser degree at some of these sites (including *Fkbp5* and *Cry2*).

### Transactivation assays demonstrate mutual inhibition at standard response elements

As our ChIP-reChIP results indicate co-binding at select sites, and a probable competition at other sites, we tested whether the two transcription factors could impact each other’s transactivation capacity at respective classical response elements. That is, we explored if LRH-1 impacted the transactivation by ERβ at the classical ERE, and reverse, if ERβ impacted the activity of LRH-1 at its NR5A-RE, using luciferase transactivation assays, each in triplicate independent experiments. Interestingly, the E2-induced ERβ transactivation of the ERE-TATA reporter construct was significantly repressed in the presence of LRH-1 (Fig. 6F and Additional file 1: Fig. S3C). Reciprocally, the LRH-1 transactivation of the NR5A-RE reporter construct was counteracted by ERβ, independently of E2 treatment (Fig. 6F and Additional file 1: Fig. S3D). Unexpectedly, the LRH-1 transactivation of the NR5A-RE reporter construct was enhanced by E2, although neither ERα nor ERβ is expressed in the cell line. These results demonstrate a negative crosstalk between ERβ and LRH-1 at their respective response element, where they repress each other’s transcriptional activity. Thus, their co-regulation of target genes may include a repressive mechanism.

## Discussion

ERβ is known to be critical for ovarian function in multiple species. The ERβKO mouse phenotype of impaired follicular maturation, with a reduced number of fully mature follicles, is correlated with smaller litters. Meanwhile, the production of fewer litters may partially be linked to dysregulated ovulatory signaling. However, most of the knowledge is based on histological observations in knockout animals or transcriptomic analysis on isolated granulosa cells [25, 26, 29, 30]. The ovarian ERβ molecular mechanism of action, including its direct target genes, remains largely unknown. Further, the in vivo molecular mechanism is intrinsically difficult to study as there is no other cell type where ERβ is highly expressed, and its expression is absent (or lost) in cultured cells. In this study, we denote the endogenous ERβ genome-wide chromatin binding in vivo (mouse) and identify those that are also transcriptionally altered as its direct ovarian target genes. Moreover, we unravel an overlap and interaction between the ERβ and LRH-1 regulatory networks that we propose is a critical ovarian mechanism that underlies female fertility.

Our transcriptomic analysis of ERβ in WT and ERβKO ovary supports a role for ERβ in ovarian lipid metabolism, as has previously been indicated in isolated granulosa cells during ovulation [29], and we reveal its impact on genes involved in glucose metabolism. Lipids are important as a source of energy for both folliculogenesis and ovulation [55], and are crucial for the steroidogenesis that takes place in granulosa cells. Further, glucose in the follicular fluid is used by the non-vascular membrane granulosa cells [56, 57]. Both lipid and glucose metabolism have been shown to be important for the cumulus-oocyte complex, where the cumulus cells provide essential metabolites for the oocyte [58, 59]. Interestingly, the digital cytometry analysis revealed a significantly increased expression of cumulus cell markers, as well as a general augmentation of overall granulosa cell population markers in ERβKO mice. This could partly explain the differential expression of genes related to lipid metabolism and glucose homeostasis after the loss of ERβ. We found genes, both directly and indirectly regulated by ERβ, that are active in cholesterol, steroid, and fatty acid metabolism. Their gene products, in addition to the enzymes that directly impact estrogen synthesis (Cyp11a1, Hsd17b1), also included proteins involved in cholesterol metabolism and steroidogenesis (e.g., Cyp27a1, Fdps, Fdxr, Hmgcs1, Lrp8, Lrp11), as well as in prostaglandin synthesis and fatty acid synthesis/elongation pathways (e.g., Ptgis, Fasn, Lep, Cds1, Hgpd, Acacb) which can impact steroidogenesis. Noteworthily, most of the DEGs belonging to the lipid pathway presented ERβ binding sites in their regulatory regions, while the DEGs involved in glucose metabolism did not contain ERβ binding sites, suggesting the latter are indirectly regulated as a consequence of lost ERβ. Further, our data confirm previous findings that *Cyp11a1* (ERβ-binding site in intron 2), *Gata4* (4 ERβ-binding sites, including in promoter), and *Runx2* (no ERβ-binding site) are regulated upon loss of ERβ in granulosa cells [29, 30]. However, we observed an upregulation rather than a downregulation of *Gata4*, and several previously identified key ERβ-regulated genes were not found to be affected in our study (incl. *Jaml*, *Ptgs2*, *Dusp9*, and *Mageb16*, which were barely detected (< 1 FPKM) and did not exhibit any ERβ-binding sites). A difference is, of course, that our analysis included the whole ovary, and our data is impacted by differences in cell composition.

It is well known that FSH promotes the development of ovarian follicles, and that LH regulates the preovulatory maturation of oocytes, ovulation, and formation of the corpus luteum. These surges also activate pro-inflammatory genes through cAMP signaling in the ovary and the secretion of proteolytic enzymes by the follicle. These enzymes degrade the follicular tissue at the site, resulting in oocyte release [60, 61]. ERβKO mice have been reported to have an improper thinning of the follicular wall and to lack a proper expansion of the cumulus-oocyte complex [26]. Our deconvolution data show that a lack of ERβ is accompanied by an increase of theca cells in the follicular wall. While fewer cumulus cells have been reported in the knockout mice [25], we observe an increased number of specific *Nupr1*^high^ (nuclear protein 1) cumulus cells. *Nupr1* has been linked to inflammatory response and has been shown to be induced in the cumulus-oocyte complex during the ovulatory process [62]. However, *Nupr1* was also recently identified in murine atretic granulosa cells [63]. As one phenotype of the ERβKO mice is an increased number of atretic large antral follicles [26], an increased number of cumulus cells expressing high levels of *Nupr1* could explain the overall phenotype of a reduced number of cumulus cells. Lastly, we also observed a trend of increased *Lyz2*^high^ macrophage cells in the ovaries of the ERβKO mice. This increase in macrophages aligns with our previous findings from colon tissue, where ERβ is anti-inflammatory and reduces macrophage infiltration [64, 65]. Our study thus contributes to understanding how ERβ impacts the ovarian cell composition, which is related to the characteristic phenotypes.

A spike in estrogen level normally occurs just before ovulation, and this provides positive feedback to the hypothalamus and pituitary, which in response will induce the LH and FSH surges. ERβKO females exhibit a reduced level of E2 at diestrus [27, 28]. This is due to the local ovarian activity of ERβ, since the surge can be rescued by implantation of ovaries from WT mice [28]. It is thus interesting that we found that several of the direct ERβ-targets are known to regulate the diestrus E2 surge (incl. *Hsd17b1*, *Hsd17b3*, *Cyp11a1*). ERβ also bound chromatin by the aromatase gene. Although its expression was not altered upon ERβ loss, we note that this regulation is expected to occur specifically at diestrus and may thus only be detected at this specific time point. Overall, since we did not compare ovaries of different estrous stages, we may have missed regulations that occur at specific stages.

In accordance with ERβKO mice exhibiting a reduced size of the LH surge, we could confirm the deregulation of numerous ovarian LH and FSH surge genes. The receptors for FSH (*Fshr*) and LH (*Lhcgr*) are both expressed in granulosa cells. They are G protein-coupled receptors and signal through cAMP-dependent and cAMP-independent mechanisms which result in activated protein kinases and downstream activation of transcription factors and other proteins. In accordance, most of the LH and FSH surge genes affected by ERβ deletion (incl. *Runx2*, *Star*, *Saa3*, *Apln*, and *Hsd17b7*) did not exhibit an ERβ-binding chromatin site and can thus be concluded to be downregulated as a consequence of the reduced surge in knockout animals rather than being direct targets of ERβ. Meanwhile, both the FSH and LH receptor exhibited ERβ binding sites (*Fshr* in intron 1, *Lhcgr* 4 sites in intron 3, exon 1, and intergenic), indicative of being direct transcriptional targets of ERβ. *Fshr* was significantly upregulated in the knockout, whereas *Lhcgr* was not regulated. Thus, *Fshr* appears to be repressed by ERβ in the ovary. However, we cannot exclude that this regulation is a feedback mechanism to compensate for the reduced surges.

One of the strongest impacted genes upon ERβ deletion was *Bhmt*. This gene is normally highly expressed in luteal cells and is known to be absent in infertile mice. In our study, *Bhmt* was consequently highly expressed in the WT ovary (35 FPKM) but was completely absent (0.3 FPKM) in the ERβKO ovary. This could simply be a consequence of the ovulatory phenotype (dramatic reduction of luteal cells), but our finding that ERβ binds intergenic chromatin located downstream (29 kb from TSS) of the gene itself, indicates a potential direct regulation. *Bhmt* expression has also been linked to the transcription factor *Cebpa* (C/EBPα). *Cebpa* mediates LH-activated ERK1/2-dependent granulosa cell differentiation and is essential for ovulation and luteinization. *Cebpa* knockout females are subfertile, similar to ERβKO females [66]. As the *Cebpa* gene was also bound by ERβ (two intergenic chromatin sites: 24 kb upstream and 6 kb downstream of TSS) and downregulated following ERβ knockout, ERβ may regulate *Bhmt* both directly and indirectly (via *Cebpa*).

On a mechanistic note, we observed that nearly half of the ERβ chromatin binding sites were located in introns. As an example, multiple low-density lipoprotein receptor-related proteins genes (*Lrp1*, *Lrp4*, *Lrp5*, *Lrp8*, *Lrp11*) all held ERβ bindings sites in intron 1. ERβ was furthermore essential for the expression of two of these (*Lrp8* and *Lrp11*). Lipoproteins in the plasma are the major source of cholesterol obtained by the ovarian theca and granulosa cells for steroidogenesis and *Lrp8* has been associated with reproductive traits in ducks (where it is exclusively located in granulosa cells) and has been suggested to regulate follicular growth [67]. Regulation of the Lrp proteins may thus impact estrogen production and fertility. Overall, the high frequency of ERβ bindings sites at early exons among the regulated genes, especially notable in the most strongly regulated genes (5 of 8, or 62%, of the most highly regulated gene targets), support that this as a mechanism rather than a random, non-functional binding. Studies in *C. elegans* have found that first introns are more conserved in length, are bound by more transcription factors, and that the transcription factors that bind first introns are largely different from those binding promoters [68]. Studies of the ERβ homolog, ERα, in human breast cancer cells have also found that ERα does not primarily bind promoter regions (approximately 3% of sites are in the proximal promoter region [69, 70]). From this, we propose that intron 1 binding, rather than promoter binding, may be a main mechanism for transcriptional activation of endogenous ERβ target genes.

Importantly, we identified the NR5A (LRH-1 and SF-1) motif as a DNA-binding element associated with ERβ in the ovary. We show that this motif was nearly as enriched among ERβ bound sequences as its own ERE motif. While it may be theoretically possible that the NR5A motif, which is an extension of a half-ERE site, can be bound by ERβ, this is not supported since the NR5A motif was not found to be enriched in ERβ ChIP-seq of other cells that do not express LRH-1 (exogenous ERβ expressed in colon or breast cancer cell lines [34]). Accordingly, by comparing our data to ChIP-seq of LRH-1 in granulosa cells from the literature, we could conclude that ERβ and LRH-1 indeed bind numerous identical sites over the genome in close vicinity of each other. Notably, among the direct ERβ targets where ERβ was essential for gene expression, five of eight were also bound by LRH-1 (*Ctnna2*, *Lrp8*, *Slc38a3*, *Cabp1*, *Tspan11*). This may suggest that a subset of granulosa cells genes require the cooperative activity of both ERβ and LRH-1 in the transcriptional complex for their expression. This connection between ERβ and LRH-1 is likely intrinsic to granulosa cells of the ovary, where both these nuclear receptors are highly expressed.

Our finding that the larger group of de facto dual-bound sequences contained only the NR5A motif and no ERE may support the hypothesis that LRH-1 binds first, and then recruits ERβ to the chromatin. This is in line with reports of LRH-1 acting as a pioneer factor in mice where it can bind nucleosomal DNA in vitro and promote chromatin accessibility during zygotic genome activation in two-cell embryos [71]. It is further possible, as schematically proposed in Fig. 6, that chromatin-bound LRH-1 recruits ERβ bound to DNA at another location and that this enables chromatin loop-formation.

Upon testing the hypothesis that they bind simultaneously, our ChIP-reChIP experiments demonstrated that both ERβ and LRH-1 could indeed bind co-operatively within the *Greb1* chromatin area that harbors dual NR5A and ERE motifs. This confirms their nearby interaction at the chromatin level. This particular gene is known to be upregulated by ERβ when introduced into other cell types (human MCF-7 cells, which do not express LRH-1), but was indicated by our data to be downregulated by ERβ in the ovary. Whether this is a cell type or species difference, or whether the presence of LRH-1 impacts the direction of regulation, needs further studies. Furthermore, the same experimentation showed that at certain other sites we could not detect any bound DNA (*Cyp11a1*) or no clear enrichment (*Fkbp5*). Our experimentation using transactivation assays showed that they can inhibit each other’s transcriptional activity at their classical response element. It is thus possible that ERβ and LRH-1 bind simultaneously at some sites, but one at a time at other sites, perhaps competing for binding. Whether one receptor’s transactivation (at its respective response element) is inhibited by the other nuclear receptor, is because they can bind each other’s motif (to be noted, the NR5A motif harbors sequences identical to an ERE half-site), or because of protein-protein interaction, remains to be investigated. Further, as sites far apart may directly interact with each other, investigating the related 3D chromatin architecture, using for example Hi-C sequencing, as well as exploring chromatin accessibility (ATAC, FAIRE, or DNase susceptibility) to show which sites lose transcriptional factor accessibility following ERβ deletion, can help to further elucidate their precise mechanism of action. As LRH-1 is essential for fertility, and its deletion leads to the formation of large preovulatory follicles with failure to ovulate, similar to the ERβKO phenotype [39], our data provide a direct link between these two fertility regulators.

Overall, the strengths of our study include the usage of a highly validated antibody, the unbiased approach of ChIP-seq and RNA-seq, the analysis of the endogenous ERβ activity within the tissue environment, and the comparison with knockout animals. To be noted, we also compared our data with ERβ ChIP-seq in ERβKO animals, and a direct comparison (WT ChIP vs ERβKO ChIP) confirmed over 70% of sites (WT ChIP vs input). We consider this a high fraction, considering the caveats of using ChIP on non-existent proteins. The accuracy of the cistrome is evidenced by the significant enrichments of the ERE and well-known associated motifs AP-1 and GATA. Moreover, a recent study of several ovarian transcription factors included ChIP-seq of ERβ. Although  this was performed with a non-validated antibody (Aviva, cat no: ARP37039, RRID: AB_10714286), without replicates nor corresponding input, it did nevertheless identify 3976 of our 4875 binding sites [72]. This supports the validity and generality of the provided cistrome. While we have previously studied exogenously expressed human ERβ with the same antibody and revealed its chromatin binding pattern in non-reproductive cell types, including breast and colon cancer cell lines [34, 43], and others have reported endogenous ERβ cistromes but not using the validated antibody (rat male germ cells [36] and mouse endometrium overexpressing ERβ [37]), this is to our knowledge the first time the endogenous ERβ cistrome is described in detail using validated tools.

Some weaknesses of the study should be highlighted. Since we analyzed non-synchronized ovaries in RNA-seq, the gene expression data is diluted by cells not expressing ERβ. This means that low-abundant ERβ-regulated genes are likely to be missed. Also, genes that are regulated at specific estrous stages will most likely not be detected. Thus, more genes are likely regulated than what is demonstrated in this study. This may explain some discrepancies between this study and the literature. Consequently, while we gained in studying the complete impact on the in vivo ovarian context, further studies are needed to comprehensively define the full spectrum of regulated genes, including at specific phases of the estrous cycle. However, it should be noted that a complete overlap between identified ChIP-binding sites and regulated genes is not expected. The identified overlap here is similar to what has been previously demonstrated for ERα in homogenous cell lines [43, 69, 73]. In addition, our study does not separate the binding and activity of the two ERβ splice variants present in the mouse ovary. Understanding how they impact chromatin binding and transcriptional activation is another interesting topic for further studies.

## Conclusions

We here provide wide-ranging insights into the endogenous ERβ gene regulatory landscape in the ovary and identify its ovarian target genes at the genome-wide level. Our work reveals mechanistic insights including that ERβ and LRH-1 have a shared cistrome and that they can bind simultaneously at some sites, and not at other sites, and that they can inhibit each other’s transcriptional activity. Altogether, we provide a foundation that enables a better understanding of ovarian physiology and female fertility. This data can support research for an effective treatment to overcome ineffective follicle development and oocyte maturation.

## Methods

### Animal experiment and tissue collection

Ovaries from mice with (WT) and without expression of ERβ (ERβKO) was used for this study (*N* = 80, age 4–20 weeks). ERβKO animals were generated by heterozygous breeding of ERβ^+/−^ mice generated from ERβ^flox/+^ mice (B6.129 × 1-Esr2^*tm1Gust*^, backcrossed on C57BL/6J) crossed with transgenic Rosa26-Cre deleter mice (Taconic) [74]. Deletion of exon 3 in the offspring was confirmed by standard PCR protocol with primers listed in Additional file 8: Table S7. This deletion results in a frameshift and premature stop codon, leading to a subsequent absence of ERβ protein (Fig. 1D-E). Control mice were offspring lacking Cre (thus WT ERβ). Animals were housed under a controlled environment at 20 °C with a 12-h light-dark cycle. Ovaries were harvested and prepared for ChIP-seq, ChIP-qPCR, ChIP-reChIP, IHC, WB, and RNA analysis as detailed for each experiment. The ovaries were not collected at a specific stage of the estrus cycle. All experiments were performed in accordance with the EU Directive 2010/63/EU for the care and use of laboratory animals. The local ethical committee of the Swedish National Board of Animal Research (Stockholm ethical committee, Dnr ID 211) approved all experimental protocols.

### Immunohistochemistry

Sections of formalin-fixed paraffin-embedded mouse ovary from WT (*n* = 14) and ERβKO animals (*n* = 8) were deparaffinized in xylene and rehydrated in decreasing concentrations of ethanol. The sections underwent heat-mediated antigen retrieval in citrate buffer (pH6, 15 min) followed by permeabilization (0.1% IGEPAL, 15 min) and blocking of endogenous peroxidase activity (3% hydrogen peroxidase in 50% methanol in PBS, 30 min). Slides were further blocked for unspecific binding of secondary antibodies (5% normal goat serum, 30 min at 4 °C) and unspecific avidin/biotin binding (DAKO). Incubation with primary anti-ERβ antibody (mouse monoclonal PPZ0506, R&D Systems Bio-Techne, cat no: PP-PPZ0506-00, lot no: A2, RRID: AB_2293861, 1:200 dilution) in 0.1% IGEPAL in PBS was performed overnight at 4 °C. Negative controls without primary antibody were used for each slide. The sections were incubated with appropriate biotin-conjugated anti-IgG secondary antibody (goat anti-mouse IgG, ThermoFisher, cat no: 31800, RRID: AB_228307, 1:500 dilution) for 1 h at room temperature (RT). Following incubation with avidin-biotin complex (ABC, ThermoFisher) for 45 min at RT, the staining was developed with Liquid DAB+ (3,3- diaminobenzidine) Substrate Chromogen System (DAKO). The slides were counterstained with Mayer’s Hematoxylin and dehydrated in ethanol and xylene. Images were captured using a BX53 light microscope and CAM-SC50 camera (Olympus, Tokyo, Japan). The ERβ staining was assessed using a combinative semiquantitative scoring of staining intensity and area.

### RNA in situ hybridization

WT ovary (*n* = 2) was fixed in 4% formaldehyde (24 h), stored in 70% ethanol, embedded in paraffin, and sectioned. The sections were used for RNA in situ hybridization with mouse *Esr2* probe (316121) using RNAScope 2.5HD Assay – Brown according to manufacturer’s protocol (RNAScope ACDBio). Mouse *Ubs* (310771) primer and *Dapb* were used as a positive and negative control, respectively, in mouse liver.

### RNA isolation

Frozen ovarian tissue from WT (*n* = 5) or ERβKO (*n* = 5) mice was homogenized with a tissue lyser (Qiagen, Chatsworth, CA). Total RNA was isolated with QIAzol and purified using the miRNeasy Mini Kit (Qiagen, Chatsworth, CA) according to the standard protocol, and on-column DNAse treatment was applied. Quantitative and qualitative analyses were performed with NanoDrop 1000 spectrophotometer and Agilent Tapestation 2200 (Agilent Technologies, Palo Alto, CA), respectively.

### cDNA synthesis and qPCR

One microgram RNA was reverse transcribed using the iScript cDNA synthesis kit (Biorad) according to standard protocol. Ten nanograms of cDNA were used to perform qPCR in a 10 μl volume, using iTaq universal SYBR Green supermix (Biorad), as recommended by the supplier. Non-template negative controls and melting curve analysis were used to ensure specific amplification. Samples were run in duplicates using the CFX96 Touch System (Biorad) and the relative gene expression was calculated as the mean per group using the ΔΔCt method, normalized to the geometric mean of two reference genes (*Actb* and *Gapdh*). All primer sequences are provided in Additional file 8: Table S7.

### Western blot

Frozen ovaries from WT (*n* = 2, 2 ovaries per replicate) and ERβKO mice (*n* = 2, 2 ovaries per replicate) were homogenized using a tissue lyser (Qiagen, Chatsworth, CA). Total protein was extracted in RIPA buffer (ThermoFisher, cat no: 89900) with 1× protease and phosphatase inhibitor, incubated for 30 min on ice, vortexed for 60 s, and centrifuged at 13,000 rpm for 15 min at 4 °C. Protein concentration was measured by BioRad Protein Assay Kit (Bio-Rad) and a UV spectrophotometer. On a 10% SDS-polyacrylamide gel (Biorad, cat no:4561034), 20 µg of total protein and ladder were loaded and electrophoresed. Using Transblot Turbo Transfer Kit and system the separated proteins were transferred to a PVDF membrane for 10 min (Bio-Rad, cat no: 170-4273). The membrane was incubated in 5% milk for 1 h at RT before incubation with primary antibodies against ERβ (PPZ0506, 1:1000) and GAPDH (ThermoFisher, cat no: MA5-15738, lot no: UH277724, RRID: AB_10977387, 1:1000) in 5% milk overnight. Membranes were washed four times with 0.1% TBST for 10 min, and then incubated with anti-mouse IgG HRP-conjugated secondary antibody (Cell Signaling, cat no: 7076, lot no: 33, RRID: AB_330924, 1:5000). Clarity western ECL substrate (Bio-Rad, cat no: 170-5061) was used to visualize the protein and the images were recorded using an imaging device (ThermoFisher).

### ERβ ChIP-seq and data analysis

Fresh WT ovaries (*n* = 14, from 7 mice, per triplicate) and ERβKO ovaries (*n* = 14, from 7 mice, per triplicate) were collected from 4–16-week-old mice (*N* = 42), washed with PBS, cut into small pieces, and cross-linked with formaldehyde (1%, 15 min, with shaking in between). Formaldehyde was removed by PBS washes and glycine (0.125 M) was added to stop further cross-linking. The samples were stored at -80 °C. Cells were separated using a Dounce homogenizer on ice, passed through a 100 µM cell strainer to remove connective tissue, washed with ice-cold PBS, and cell pellets were collected by centrifugation at 4500 rpm. At 4 °C, with ice-cold reagents and incubations on shaking, cell pellets were lysed (10 min) in LB1 (50 Mm HEPES, 140 mM NaCl, 1 mM EDTA, 10% glycerol, 0.5% IGEPAL and 0.25% Triton-X) and centrifuged (4500 rpm, 5 min). The pellets were then resuspended (5 min) in LB2 (10 mM Tris-HCl, 200 mM NaCl, 1 mM EDTA), centrifuged, and dissolved in LB3 buffer (10 mM Tris-HCl, 100 mM NaCl, 1 mM EDTA, 0.5 mM EGTA, 0.1% Na-deoxycholate and 0.5% Na-lauroylsarcosine) and sonicated to generate 200–500 bp chromatin fragments. Chromatin samples were centrifuged at 13,000 rpm (5 min), and supernatants were transferred into low-binding DNA tubes and incubated overnight with ERβ antibody (PPZ0506) or IgG (mouse polyclonal, Santa Cruz, cat no: sc-2025, lot no: J1514, RRID: AB_737182) as a control. After overnight incubation, 30 µl of protein G Dynabeads were added (cat no: 10004D, Invitrogen) and incubated (4 h). Beads were washed with TSE1 (20 mM Tris-HCl, 150 mM NaCl, 2 mM EDTA, 0.1% SDS and 0.1% Triton-X), followed by TSE2 (20 mM Tris-HCl, 500 mM NaCl, 2 mM EDTA, 0.1% SDS and 1% Triton-X), LiCl buffer (20 mM Tris-HCl, 1 mM EDTA, 250 mM LiCl, 1% IGEPAL and 1% Na-deoxycholate) and TE buffer (10 mM Tris-HCl and 1 mM EDTA) for 10 min in each buffer. Samples were eluted with a NaHCO3 (0.75%) buffer containing SDS (1%) and proteinase K. Cross-linking was reversed overnight at 65 °C, followed by treatment with RNase A (1 h, 37 °C) and DNA purification using QIAquick PCR purification columns (Qiagen, cat no: 28104). The resulting DNA was used to prepare ChIP-seq libraries as using the NEB Next Ultra II DNA Library Prep Kit as previously described [33, 34], and sequenced on NextSeq 550 (75 cycles single read) with the V2 reagent kit (Illumina) at the Bioinformatics and Expression Analysis (BEA) core center at Karolinska Institute. To be noted, an IgG control ChIP was performed, but it could not be used as it did not recover enough DNA to build a library. STAR was used to map ChIP-seq reads to the mouse reference genome assembly GRCm38 (mm10) with the alignIntronMax flag set to 1. Only uniquely mapped reads were used for downstream analysis. HOMER was used for peak calling, applying a cut-off of false discovery rate (FDR) < 0.001 and > 4-fold enrichment over control input. Peaks overlapping within 200 bp were merged, and only peaks present in at least two biological replicates were considered for further analysis. De novo motif analysis was performed using HOMER default parameters. The motif sequences were further scanned in HOMER using annotatePeaks.pl for the estrogen response element (ERE: GGTCASNBTGAC), LRH-1 (CYDTGACCTTGA), and GATA (BNWGATAA), which unlike de novo motif analysis also considers half and putative motifs. The raw tag counts were normalized in R, and the edgeR package was used to identify differences in binding patterns. A complex heatmap was used to cluster and visualize the peaks. The data is available at GEO (GSE203391, [75]). Gene Ontology/biological function analysis of the genes located nearest to the binding sites was performed using DAVID.

### RNA-seq and bioinformatic analysis

Total RNA (300 ng, RNA integrity number (RIN) > 8) from frozen ovaries from WT (*n* = 5) or ERβKO (*n* = 4) mice was used for library preparation (Illumina TruSeq Stranded mRNA) and sequenced (Illumina NovaSeq6000, flow cell S4-300) at Sweden’s National Genomics Infrastructure (NGI) facility. The sequencing generated at least 13 million paired-end reads (2 × 150 bp) per sample, which were mapped to the mouse genome (GRCm38) using STAR. Gene counts and FPKM values were generated with FeatureCounts and StringTie. DESeq2, with raw counts as input, was used to calculate differentially expressed genes (DEG). The FDR was estimated by the Benjamini-Hochberg procedure. The differential expression of genes was considered significant if FDR < 0.05 and log_2_FC >|0.4|. Only genes with an FPKM average > 1 in at least one group were used for downstream analysis. The data is available at GEO (GSE196650, [76]). Analysis of gene ontology/biological function was carried out using the DAVID bioinformatics website, and the REVIGO web server [77] was used to cluster the terms based on a clustering algorithm to find representative subsets of terms. GOplot (version 1.0.2) was used in R (version 4.1.0) to visualize gene expression and GO terms in a GOcircle plot.

### Digital cytometry

CIBERSORTx [45] was used to estimate the abundances of member cell types based on our bulk ovary RNA-seq sequencing data with default settings. The annotated ovary single-cell expression matrix from a single-cell mouse cell atlas (scMCA) [44] was used as a reference. Transcripts Per Million (TPM) normalization was performed for both the single cell reference and bulk RNA-seq counts before the CIBERSORTx processing.

### ChIP-reChIP of ERβ—LRH-1

ChIP-reChIP was performed in duplicates, using ovaries from WT mice (*N* = 14, using 7 mice/14 ovaries per replicate). First ERβ was ChIP:ed onto protein G Dynabeads as performed to the ChIP-seq (above). Following the washing steps (with TSE1, TSE2, LiCl buffer, and TE buffer), the ERβ-bound chromatin complexes were eluted with 0.1 M citrate buffer (pH 3) into low-binding DNA tubes. This was incubated overnight with LRH-1 antibody (mouse monoclonal PPH2325, R&D Systems Bio-Techne, cat no: PP-H2325-00, lot no: A2, RRID: AB_2154053). An additional negative control without LRH-1 antibody was included. Next, following incubation with protein G Dynabeads and washing (TSE1, TSE2, LiCl and TE buffers), the samples were eluted per the ERβ ChIP protocol. The resulting DNA was used in qPCR to amplify sites predicted to be bound by both ERβ and LRH-1 (*Greb1*, *Fkbp5*, *Cyp11a*) or only ERβ (*Cry2*). ChIP-reChIP results were depicted both as fraction of input as well as the fraction of ERβ ChIP. Primers of a DNA desert were used as negative control. Primer sequences are provided in Table S7. An ERβ ChIP from WT mice (*N* = 4, 8 ovaries pooled) was repeated as confirmation of ERβ binding (as above for ChIP-seq). The result is normalized to input and illustrated as fold change over negative control.

### Cell culture and transactivation luciferase assay

SW480 cells (authenticated with SNP profiling and tested for mycoplasma) were used for the transactivation luciferase assay. The cells, which do not express ERβ nor LRH-1, were cultured in DMEM-high glucose (Merck) with 10% fetal bovine serum (FBS), 1% penicillin-streptomycin (Invitrogen), and 2 mM L-Glutamine at 37 °C with 5% CO_2_. On day 1, a total of 2 × 10^5^ cells per well was seeded in 24-well plates in complete medium. On day 2, the cell medium was changed to media without antibiotic 1 h prior to transfection. The cells were transfected with 250 ng of pcDNA-ERβ, pcDNA-LRH1, or pcDNA-empty expression vector per well using Xtreme-GENE DNA HP (Roche) in Opti-MEM. After 5 h the medium was replaced by fresh complete medium without antibiotics. On day 3, 500 ng of either ERE-TATA-LUC [65] or NR5A-RE-LUC (cloned from the human SHP promoter) [78] reporter plasmid per well were transfected. After 5 h the medium was replaced by Phenol red-free DMEM medium (Merck) supplemented with 5% DCC FBS (ThermoFisher Scientific) containing either DMSO (vehicle) or 10 nM E2. Whole cell lysate was harvested 24 h later, and luciferase activity was measured using the Dual-Luciferase Reporter Assay kit (Promega) in a multimodal microplate reader with an injector (HIDEX SENSE). Renilla luciferase was used as an internal control. Three independent experiments were performed.

### Statistical analysis

GraphPad Prism was used for statistical analysis (GraphPad Software Inc, La Jolla, CA). The results are expressed as mean ± SEM. For the luciferase reporter assay, a two-way analysis of variance (ANOVA) was used for multiple comparisons between the different conditions followed by Fisher’s LSD test. A *p-*value < 0.05 was considered statistically significant (*p < 0.05, **p < 0.01, ***p < 0.001).

### Supplementary Information

**Additional file 1: Fig. S1.** Integrative genomic viewer showing the transcripts of *Esr2 *detected in the ovarian RNA-seq of WT mice (*n* = 5). The red box marks the insert region of isoform 1 (ERβ_ins) in the ovary of WT mice. **Fig. S2.** Additional ChIP-seq results and comparisons. Venn diagrams illustrating (A) detected ERβ-binding sites when normalizing ERβ ChIP-seq triplicates of WT ovaries against ERβ ChIP-seq of ERβKO ovaries, and (B) comparison of results when normalizing against input versus normalizing against ChIP-seq of ERβKO ovaries. (C) Enriched motifs among ERβ chromatin-binding sites in promoter (−1 kb to +100) and enhancer regions (−50 kb to +2 kb), respectively. (D) Venn diagram representing the overlap of our ERβ ChIP-seq and whole ovary RNA-seq of WT and ERβKO mice, compared with microarray of isolated granulosa cells from WT and ERβKO ovaries before and after ovulatory signal [29], with ERβ bound and regulated genes displayed in bold and red, respectively. **Fig. S3.** Additional replicates of ERβ-LRH-1 ChIP-reChIP and the luciferase reporter assay. (A) Replicate 2 of ERβ-LRH-1 ChIP-reChIP normalized against ERβ-ChIP, and (B) replicate 1 and 2 normalized against input. Additional replicates of (C) ERE-TATA and (D) NR5A-RE luciferase reporter assay in SW480 cells.

**Additional file 2: Table S1.** Statistics of the ERβ ChIP-seq.

**Additional file 3: Table S2.** All ChIP ERβ-binding sites.

**Additional file 4: Table S3.** All differentially expressed genes between WT and ERβKO ovaries, and the enriched biological functions.

**Additional file 5: Table S4.** ERβ-binding sites by nuclear receptors.

**Additional file 6: Table S5.** Conserved ERβ-binding sites between mouse and human.

**Additional file 7: Table S6.** Common ERβ and LRH-1 binding sites.

**Additional file 8: Table S7.** Primer sequences.

**Additional file 9.** Images of original uncropped blots of ERβ protein detection.

**Additional file 10.** Supporting data values for figures where n < 6.

## Data Availability

All data generated or analyzed during this study are included in this published article, its supplementary information files, and publicly available repositories. The sequencing data generated in this study have been deposited in the NCBI Gene Expression Omnibus database under accession numbers GSE203391 (ChIP-seq, [75]) and GSE196650 (RNA-seq, [76]). Previously published data analyzed in this study can be found in the NCBI Gene Expression Omnibus database under accession numbers GSE149979 (ERβ ChIP-seq in human cell lines, [34, 47]), GSE44651 (microarray on WT and ERβKO ovaries, [29, 46]), and GSE119508 (LRH-1 ChIP-seq, [51, 52]). Uncropped blots and supporting data values for figures (where n < 6) is provided in Additional files 9 and 10.

## Abbreviations

aa: Amino acid
bp: Base pair
ChIP-seq: Chromatin immunoprecipitation sequencing
CYP19A1: Aromatase
DEG: Differentially expressed gene
E1: Estrone
E2: 17β-estradiol
ERE: Estrogen response element
ERα/β: Estrogen receptor alpha/beta
ERβKO/BERKO: ERβ knockout
FAIRE-seq: Formaldehyde-Assisted Isolation of Regulatory Elements sequencing
Fbn2: Fibrillin 2
FDR: False discovery rate
FPKM: Fragments Per Kilobase of transcript per Million mapped reads
FSH: Follicle-stimulating hormone
GPER1: G protein-coupled estrogen receptor 1
Gsg1l: Germ cell-specific gene 1-like
IHC: Immunohistochemistry
LH: Luteinizing hormone
LRH-1: Liver homolog 1
Nupr1: Nuclear protein 1
RIN: RNA integrity number
RNA-seq: RNA-sequencing
scMCA: Single-cell mouse cell atlas
SF-1: Steroidogenic factor 1
TPM: Transcripts Per Million
TSS: Transcription start site
TTS: Transcription termination site
WB: Western blot
WT: Wild-type
