# Supplementary material for: Ovarian ERβ cistrome and transcriptome reveal chromatin interaction with LRH-1
Source: BMC Biol. 2023 Nov 29;21:277. doi: 10.1186/s12915-023-01773-1 (PMC10688478; doi:10.1186/s12915-023-01773-1)
Supplement: Supplementary file 1 — Additional file 1: Fig. S1. Integrative genomic viewer showing the transcripts of Esr2 detected in the ovarian RNA-seq of WT mice (n = 5). The red box marks the insert region of isoform 1 (ERβ_ins) in the ovary of WT mice. Fig. S2. Additional ChIP-seq results and comparisons. Venn diagrams illustrating (A) detected ERβ-binding sites when normalizing ERβ ChIP-seq triplicates of WT ovaries against ERβ ChIP-seq of ERβKO ovaries, and (B) comparison of results when normalizing against input versus normalizing against ChIP-seq of ERβKO ovaries. (C) Enriched motifs among ERβ chromatin-binding sites in promoter (−1 kb to +100) and enhancer regions (−50 kb to +2 kb), respectively. (D) Venn diagram representing the overlap of our ERβ ChIP-seq and whole ovary RNA-seq of WT and ERβKO mice, compared with microarray of isolated granulosa cells from WT and ERβKO ovaries before and after ovulatory signal [29], with ERβ bound and regulated genes displayed in bold and red, respectively. Fig. S3. Additional replicates of ERβ-LRH-1 ChIP-reChIP and the luciferase reporter assay. (A) Replicate 2 of ERβ-LRH-1 ChIP-reChIP normalized against ERβ-ChIP, and (B) replicate 1 and 2 normalized against input. Additional replicates of (C) ERE-TATA and (D) NR5A-RE luciferase reporter assay in SW480 cells. [file 12915_2023_1773_MOESM1_ESM.pdf]

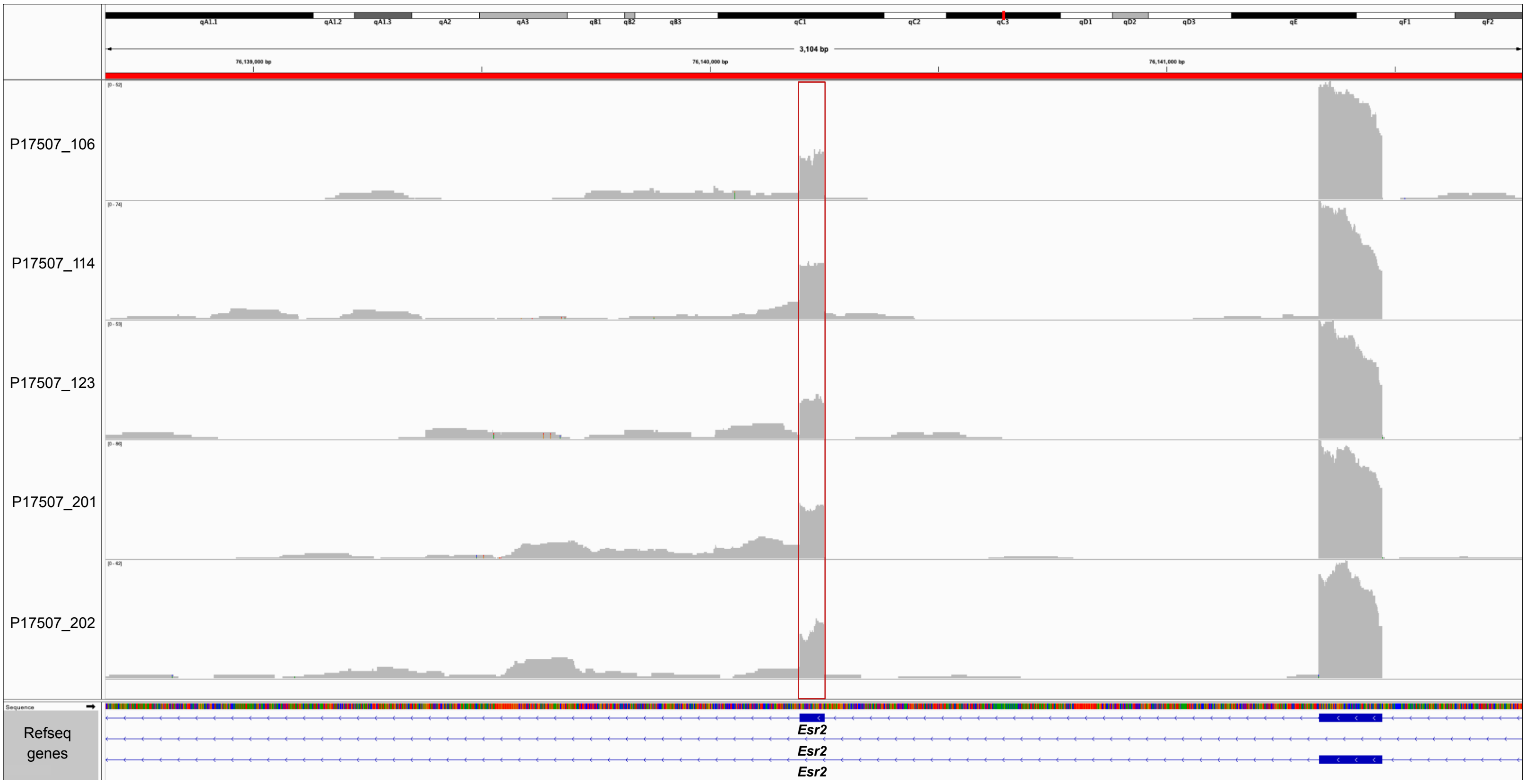

Fig. S1. Integrative genomic viewer showing the transcripts of *Esr2*. The red box marks the insert region of isoform 1 (ERβ_ins) in the ovary of WT mice.

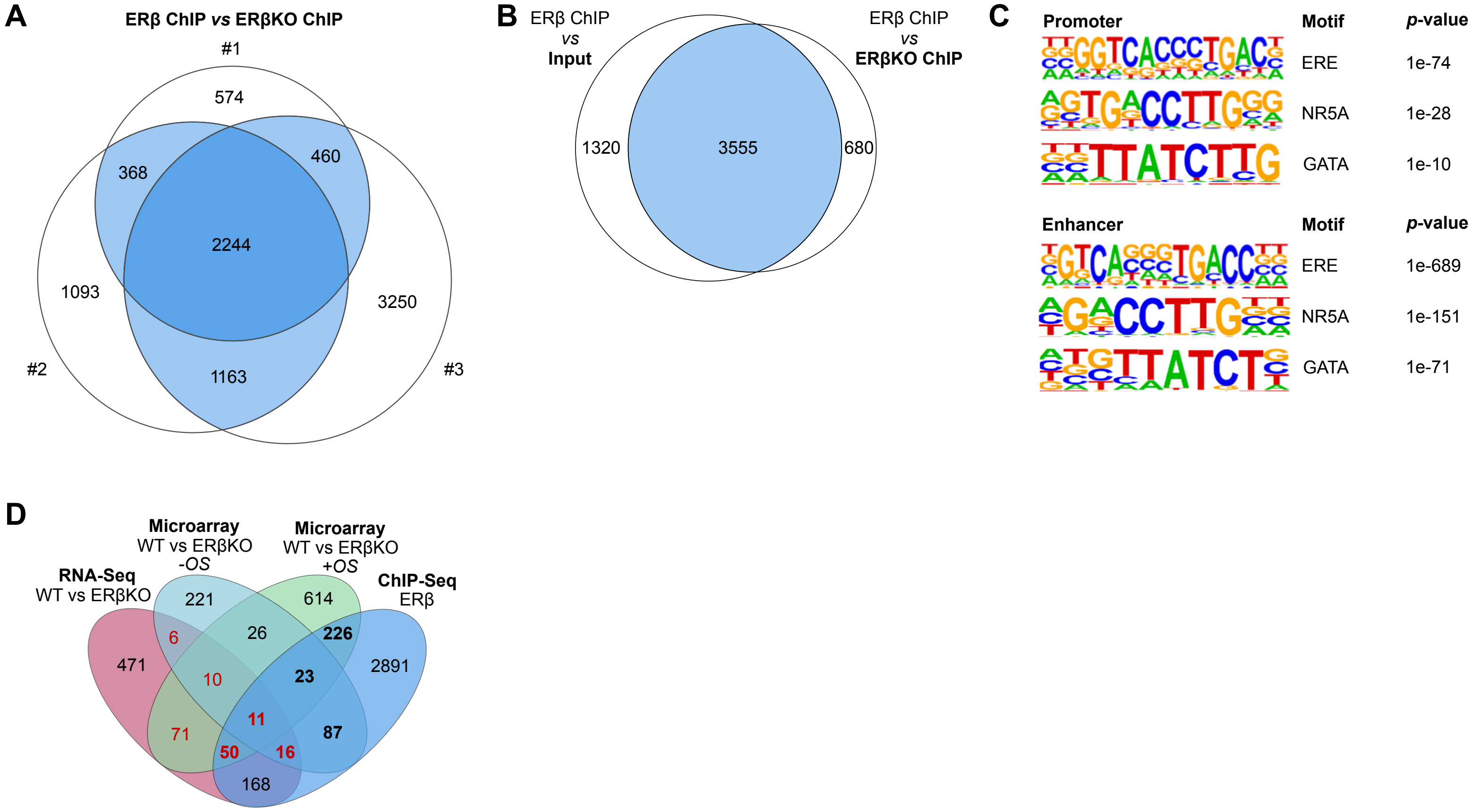

Fig. S2. Venn diagrams illustrating **(A)** detected ERβ-binding sites when normalizing ERβ ChIP-seq triplicates of WT ovaries against ERβ ChIP-seq of ERβKO ovaries, and **(B)** comparison of results when normalizing against input versus normalizing against ChIP-seq of ERβKO ovaries. **(C)** Enriched motifs among ERβ chromatin-binding sites in promoter (−1 kb to +100) and enhancer regions (−50 kb to +2 kb), respectively. **(D)** Venn diagram representing the overlap of our ERβ ChIP-seq and whole ovary RNA-seq of WT and ERβKO mice, compared with microarray of isolated granulosa cells from WT and ERβKO ovaries before and after ovulatory signal (28), with ERβ bound and regulated genes displayed in bold and red, respectively.

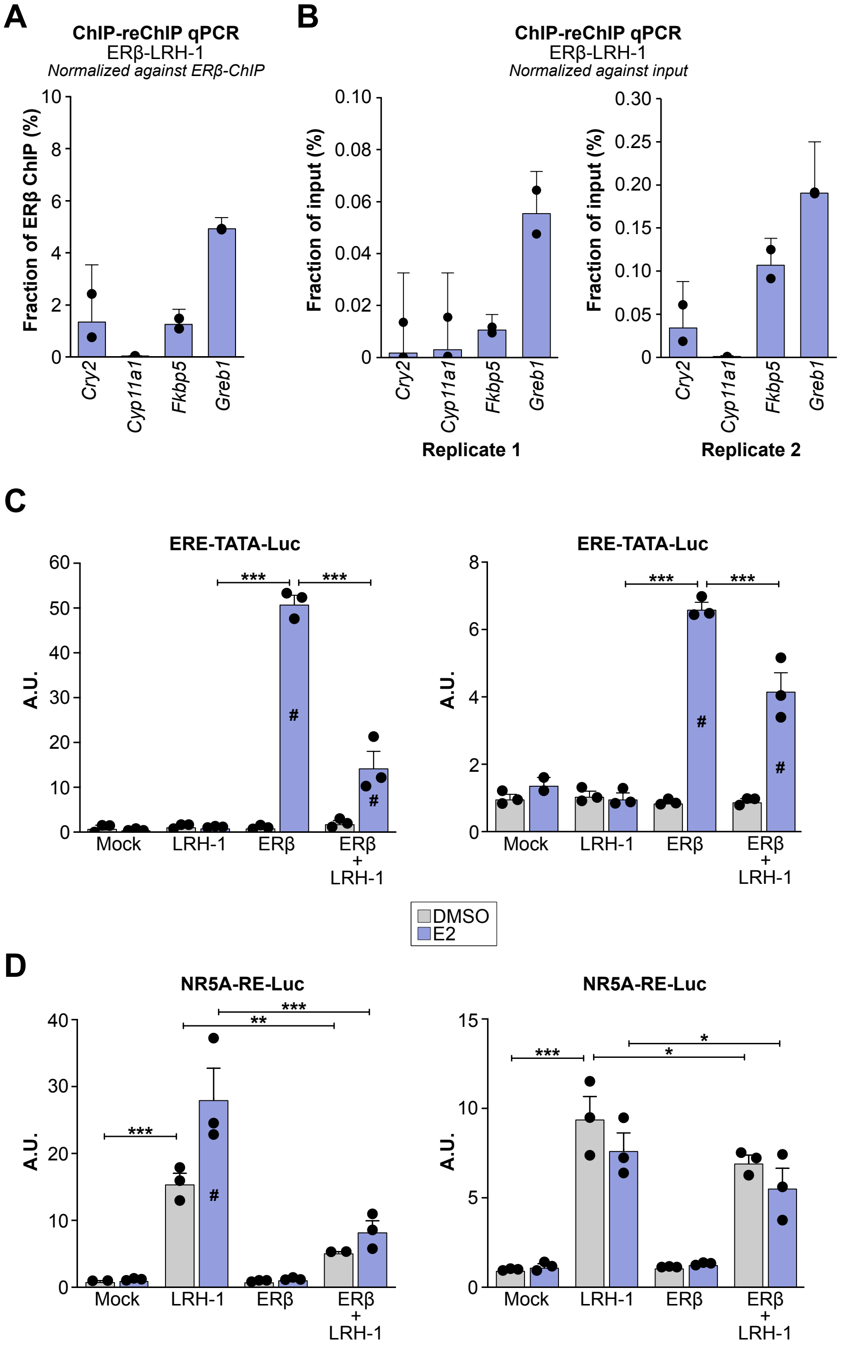

Fig. S3. Additional replicates of ERβ-LRH-1 ChIP-reChIP and the luciferase reporter assay. **(A)** Replicate 2 of ERβ-LRH-1 ChIP-reChIP normalized against ERβ-ChIP, and **(B)** replicate 1 and 2 normalized against input. Additional replicates of **(C)** ERE-TATA and **(D)** NR5A-RE luciferase reporter assay in SW480 cells.
