# Supplementary material for: Ovarian ERβ cistrome and transcriptome reveal chromatin interaction with LRH-1
Source: BMC Biol. 2023 Nov 29;21:277. doi: 10.1186/s12915-023-01773-1 (PMC10688478; doi:10.1186/s12915-023-01773-1)
Supplement: Supplementary file 9 — Additional file 9. Images of original uncropped blots of ERβ protein detection. [file 12915_2023_1773_MOESM9_ESM.pdf]

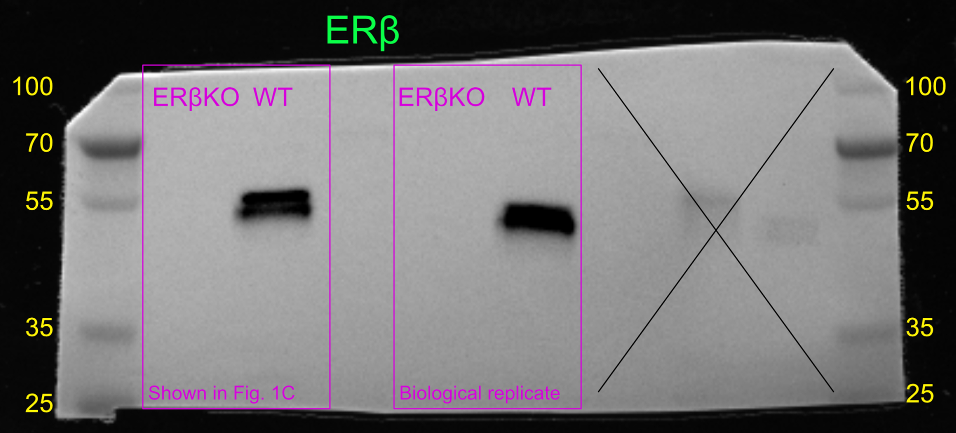
ERβ
100
70
55
35
25
ERβKO WT
Shown in Fig. 1C
ERβKO WT
Biological replicate
100
70
55
35
25

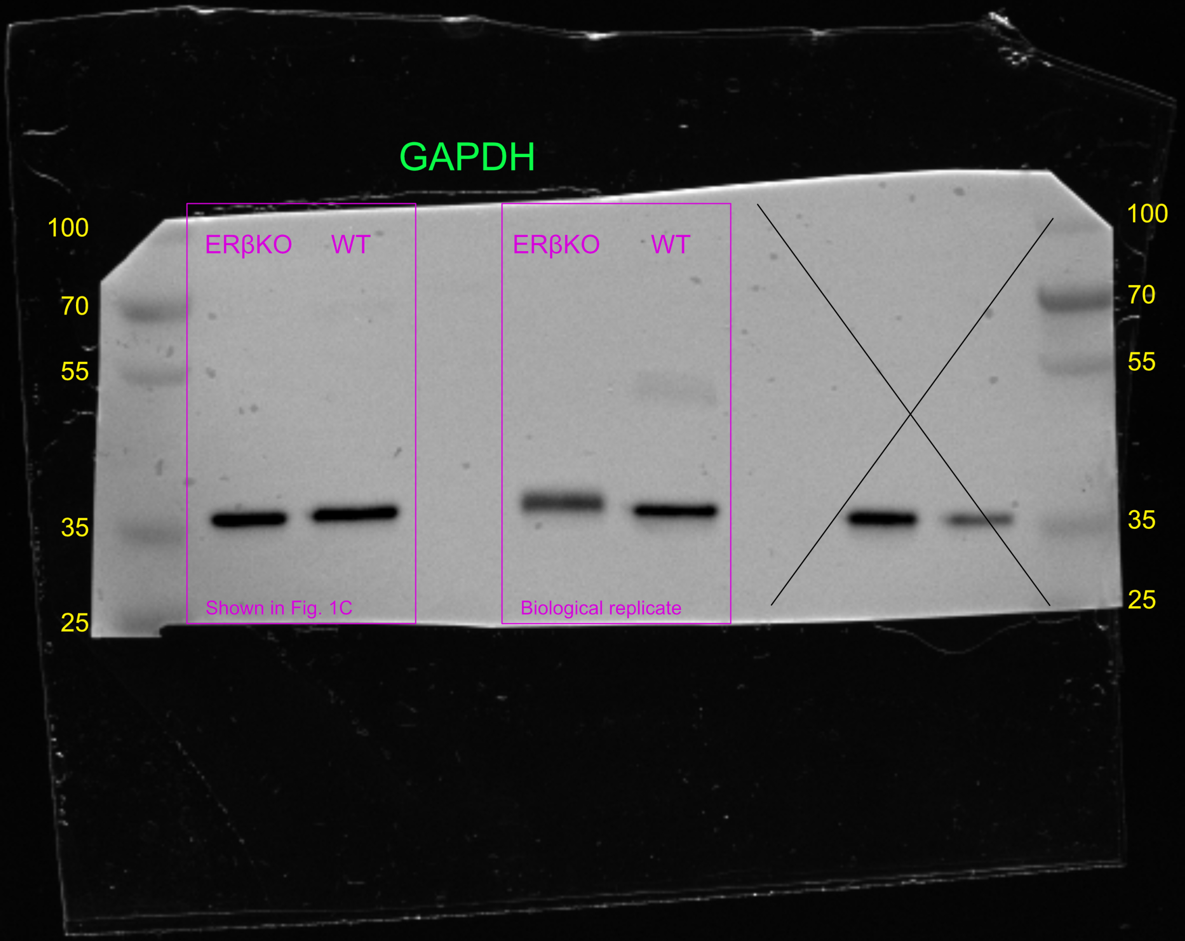
GAPDH
100
70
55
35
25
ERβKO
WT
ERβKO
WT
Shown in Fig. 1C
Biological replicate
100
70
55
35
25
